# Dual-Functional Plasmonic Z‑Scheme Bi_2_WO_6_/Ag/CuS Ternary Nanocomposite for Photocatalytic Oxidation of Tetracycline and Reduction of Cr (VI) under Visible Light

**DOI:** 10.1021/acsomega.6c05796

**Published:** 2026-09-18

**Authors:** Girija Shankar Jena, Rajakumar Ananthakrishnan

**Affiliations:** Department of Chemistry, Green Environmental Materials & Analytical Chemistry Laboratory, 30133 Indian Institute of Technology, Kharagpur 721302, India

## Abstract

The simultaneous removal of antibiotic pollutants and toxic heavy metals from wastewater remains challenging because of limited visible-light utilization and rapid recombination of photogenerated charge carriers in conventional photocatalysts. Integrating plasmonic nanostructures into semiconductor heterojunctions provides an attractive route to simultaneously enhance light harvesting and photocatalytic redox activity. In this work, a plasmonic Bi_2_WO_6_/Ag/CuS ternary nanohybrid was fabricated through sequential photodeposition of Ag nanoparticles onto hierarchical Bi_2_WO_6_ microspheres, followed by the in situ growth of CuS. The incorporation of Ag nanoparticles promotes interfacial charge transfer and enhances visible-light harvesting through the surface plasmon resonance effect. The photocatalytic activity of the ternary nanohybrid was assessed through tetracycline (TC) oxidation and Cr (VI) reduction under visible-light irradiation. The optimized ternary nanohybrid achieved 92% TC degradation and 90% Cr (VI) reduction within 60 min, corresponding to apparent rate constants of 0.0336 and 0.0322 min^–1^, respectively. Furthermore, enhanced removal efficiencies were observed in the coupled TC/Cr (VI) system, indicating a synergistic interaction between oxidation and reduction processes. Band-structure analysis and in situ EPR measurements verified the Ag-mediated all-solid-state Z-scheme charge-transfer pathway. LC–MS analysis and phytotoxicity assessment revealed the progressive degradation of TC into less toxic intermediates. The photocatalyst also exhibited good stability and considerable activity in real water matrices, highlighting its potential for practical solar-driven wastewater remediation.

## Graphical abstract

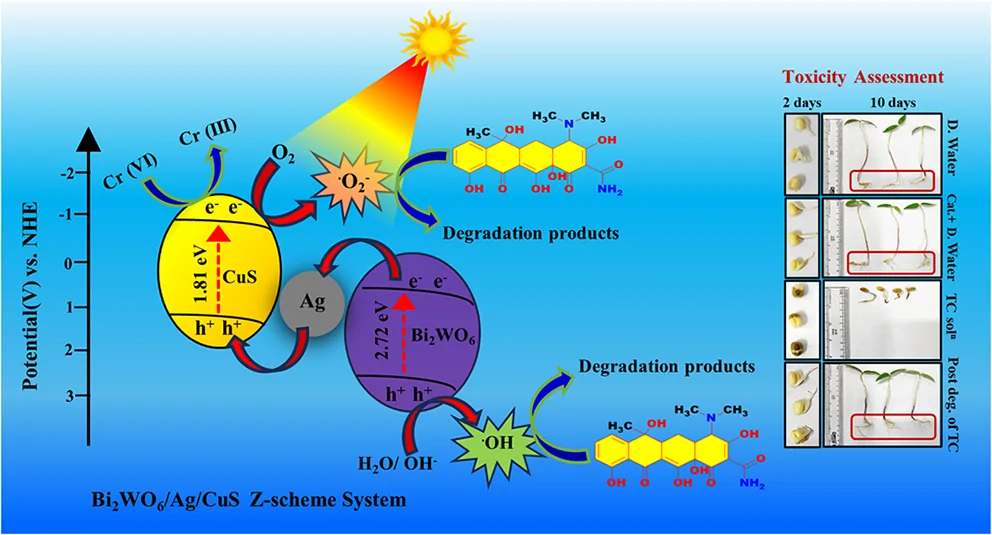

## 1 Introduction

Semiconductor-assisted heterojunction photocatalysts are essential for enhancing solar energy conversion into chemical energy owing to their ability to facilitate rapid charge separation. These heterojunctions, formed at the interface between two dissimilar crystalline semiconductors, play a significant role in various applications, including hydrogen evolution, pollutant degradation, and solar cells.^1,2^ The effectiveness of these systems depends on three key factors: light absorption capacity, charge separation efficiency, and effective charge migration for redox reactions. Designing heterostructures that optimize these aspects is crucial for developing ideal photocatalysts.^3,4^ Globalization and industrialization have led to significant environmental challenges, including water pollution caused by the overuse of antibiotics such as tetracycline hydrochloride, penicillin, and sulfamethoxazole, as well as the improper disposal of heavy metals ions like Hg^2+^, Cd^2+^, Pb^2+^, and Cr^6+^. These pollutants pose serious risks to all forms of life due to their carcinogenic, teratogenic, and mutagenic properties.^5–9^ In this context, researchers are aiming to develop semiconductor-based photocatalysts as a green approach to address these environmental problems. Our research group focuses on the preparation of visible light active semiconductor-based photocatalysts for the environmental remediation of persistent organic pollutants (POPs), dyes, and heavy metals in water and wastewater.^10,11^ Among the various semiconductors studied for photocatalysis, TiO_2_, and ZnO are promising candidates due to their nontoxic nature, affordability, and excellent stability. However, their limited efficiency under visible light restricts their broader usage in photocatalysis.^12^ Thus, it is vital to develop modified heterojunction photocatalysts for utilizing visible light and to offer enhanced performance in environmental remediation.

In the recent past, Bi-based photocatalysts have attracted a lot of attention especially because of their remarkable ability to respond to visible light, nontoxic nature, and their chemical stability. A wide array of bismuth materials, such as Bi_2_WO_6_, BiVO_4_, Bi_2_Sn_2_O_7_, BiO_2_–x, and BiOX (where X = Cl, Br, and I), have procured significant success in the domain of organics degradations.^13–17^ Their photocatalytic behavior mainly arises from the hybridization between the Bi 6s orbital and the O 2p orbital at the valence band edge, which results in a suitable band gap for visible-light absorption. Consequently, these materials emerge as up-and-coming candidates for photocatalytic applications.^18^ Bi_2_WO_6_ is an effective bismuth containing metal oxide for photocatalytic applications, attributed to its narrow bandgap of 2.7 eV, stability under light exposure, and eco-friendliness. However, its photocatalytic efficiency is compromised by rapid charge recombination and slow electron transfer processes.^2,19,20^ To address these issues, hybrid or multi-component photocatalysts featuring heterojunctions, p–n junctions, or Schottky junctions are being developed to enhance charge transport and minimize recombination. Transition metal sulfides like MoS_2_, CdS, and CuS exhibit notable electrical, optical, and catalytic characteristics.^21–23^ Specifically, CuS, recognized for its nontoxic and economical nature as a p-type semiconductor, has been integrated with Bi_2_WO_6_ to create a p-n heterojunction that enhances performance.^24^ However, the redox potential of electrons and holes is diminished during transfer in traditional heterojunctions (type-I, type-II, and p-n heterojunction) processes, which negatively impacts the efficiency of photocatalytic reactions.^25^ The Z-scheme charge transfer strategy has attracted significant attention because it preserves strong redox capability while promoting efficient charge separation. In this mechanism, low-energy charge carriers recombine at the interface, while highly energetic electrons and holes are retained to drive reduction and oxidation reactions, respectively.^26^ Z-scheme photocatalytic systems can be classified as either direct or indirect depending on the involvement of electron mediators. Several direct Z-scheme photocatalytic systems, such as BiVO_4_/WO_3_, FAPbBr_3_/Bi_2_WO_6_ and Ag_2_CrO_4_/g-C_3_N_4_,^27–29^ have shown better performance compared to individual photocatalysts. However, in certain semiconductor heterojunctions, the conventional transfer of charges may compete with the Z-scheme mechanism. In the past three decades, artificial Z-scheme systems have been developed, inspired by natural photosynthesis processes. The indirect Z-scheme incorporates electron mediators, which may be noble or nonmetal mediators, to enhance charge separation. Nobel metals generally enhance electron transfer through localized surface plasmon resonance (LSPR) and help mitigate traditional semiconductor bandgap limitations.^11,26,30^ It has been reported that Ag NPs enhance electron density and photocatalytic performance when combined with Bi_2_WO_6_.^31^ Zhu et al. showed that Ag NPs in a hybrid Ag/Fe_3_O_4_/g-C_3_N_4_ photocatalyst improves light absorption and activity, highlighting the role of LSPR in creating efficient Z-scheme photocatalysts.^32^ Reactive oxygen species (ROS) formation is an important parameter that governs various photocatalytic processes. Unlike binary systems such as Bi_2_WO_6_/CuS and BiVO_3_/WO_3_,^24,27^ typically generate hydroxyl radicals (^•^OH), while systems like FAPbBr_3_/Bi_2_WO_6_ and Bi_2_WO_6_/Ag,^28,31^ primarily produce superoxide radicals (^•^O_2_^–^). However, limited studies focus on systems generating multiple ROS simultaneously. This work aims to design a Z-scheme ternary heterojunction to facilitate the simultaneous production of numerous ROS, enhancing redox efficiency.

In this study, a hierarchical Bi_2_WO_6_/Ag/CuS Z-scheme composite was fabricated through a facile two-step wet-chemical strategy. In this system, Ag nanoparticles act as electron-transfer mediators and plasmonic sensitizers, which facilitate the recombination of electrons from Bi_2_WO_6_ with holes from CuS. Moreover, the strong affinity of Ag toward sulfur promotes the effective deposition of CuS on the Bi_2_WO_6_ surface.^33^ The structural, physicochemical, and optoelectronic characteristics of the resulting composite were systematically examined using various analytical techniques. The photocatalytic activity of the material was assessed through the degradation of tetracycline (TC) in aqueous solution under visible-light irradiation. Furthermore, the ternary composite was also employed for the reduction of Cr (VI) under optimized experimental conditions. The degradation intermediates produced during TC transformation were carefully identified, and the photocatalytic reaction mechanism was proposed based on the combined experimental and characterization results.

## 2 Experimental Section

All chemicals used in this study were of analytical grade and were used as received without further purification. Details of the chemicals and their suppliers are provided in the Supporting Information.

### 2.1 Synthesis of Bi_2_WO_6_ (BWO)

BWO was prepared via a hydrothermal method. Initially, bismuth nitrate (970 mg, 2 mmol) was dissolved in 10 mL of ethylene glycol under continuous stirring for 30 min to obtain solution A. Separately, Na_2_WO_4_·2H_2_O (330 mg, 1 mmol) was dissolved in 20 mL of deionized water to form solution B. Solution B was then added dropwise into solution A under vigorous stirring to obtain a homogeneous mixture. The resulting suspension was transferred into a 60 mL Teflon-lined stainless-steel autoclave and maintained at 160 °C for 24 h. After cooling to room temperature, the precipitate was collected by centrifugation and repeatedly washed with deionized water followed by ethanol to remove residual impurities. Finally, the obtained product was dried overnight at 60 °C to yield the BWO powder.

### 2.2 Synthesis of CuS

In a typical hydrothermal synthesis, Cu­(NO_3_)_2_·3H_2_O (1.208 g, 5 mmol) was dissolved in 40 mL ethanol and magnetically stirred for 30 min to obtain a homogeneous solution. Subsequently, Na_2_S_2_O_3_·5H_2_O (1.24 g, 5 mmol) was added to the solution under continuous stirring. The resulting mixture was transferred into a 60 mL Teflon-lined stainless-steel autoclave and heated at 90 °C for 4 h. After cooling to room temperature, the precipitate was collected by centrifugation and washed several times with deionized water and ethanol. Finally, the obtained product was dried at 60 °C for 12 h to obtain the CuS powder.

### 2.3 Synthesis of Bi_2_WO_6_@Ag (BWO/Ag)

The BWO/Ag hybrids were synthesized using a photoreduction technique. A specific amount of AgNO_3_ and 200 mg of prepared Bi_2_WO_6_ were typically put into a 20 ml methanol solution (20 Vol %). The suspension was continuously stirred for 2 h under visible-light irradiation using a 250 W lamp. The product was centrifuged, followed by washing and drying at 60 °C. The resulting samples were labeled BWO/2% Ag, BWO/4% Ag, BWO/6% Ag, and BWO/10% Ag, corresponding to mass ratio of Ag to BWO were 2%, 4%, 6%, and 10% respectively.

### 2.4 Synthesis of BWO/Ag/CuS

The BWO/Ag/CuS composite materials were synthesized via a hydrothermal approach. Initially, a specified quantity of the pre-synthesized BWO/Ag powder was dispersed in 30 mL of ethanol and magnetically stirred for 30 min to obtain a uniform suspension. Subsequently, 0.2 mmol of Cu­(NO_3_)_2_·3H_2_O and 0.2 mmol of Na_2_S_2_O_3_·5H_2_O were introduced into the dispersion under continuous stirring for an additional 30 min. The resulting mixture was then transferred into two 50 mL Teflon-lined stainless-steel autoclaves and maintained at 90 °C for 4 h. After completion of the reaction, the precipitate was separated by centrifugation, thoroughly washed with deionized water and ethanol, and dried at 60 °C for 12 h. The obtained samples were denoted as x BWO/Ag/CuS, where x represents the mass percentage of CuS in the composite (x = 5%, 10%, and 15%). For comparison, BWO/10% CuS was prepared following the same procedure using pristine BWO nanoparticles instead of BWO/Ag. Furthermore, for clarity in subsequent discussions, BWO/Ag and BWO/Ag/CuS refer to the optimized compositions of BWO/4% Ag and BWO/4% Ag/10% CuS, respectively. A schematic representation of the synthesis procedure for the BWO/Ag/CuS photocatalyst is provided in Scheme 1.

**Scheme 1:**
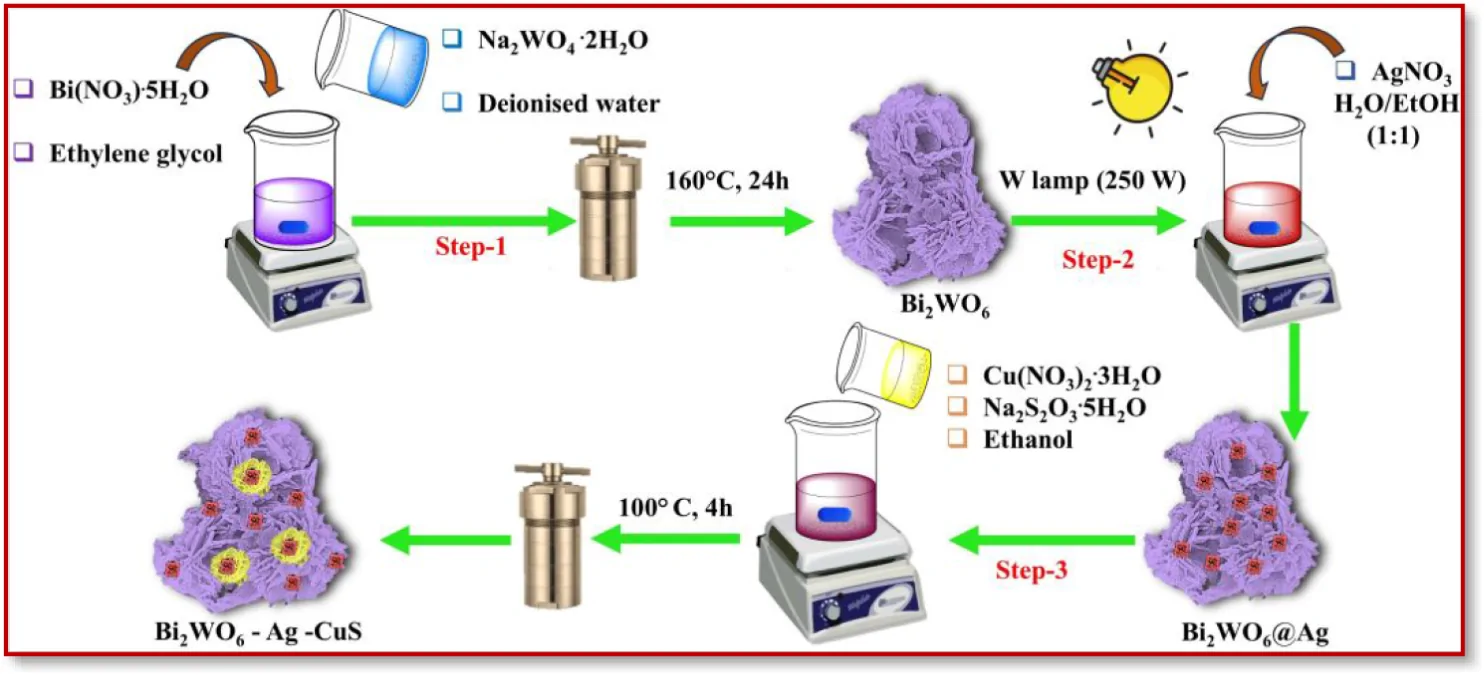
Graphical Representation of the Synthesis of a Sample

### 2.5 Characterization of Materials

Powder X-ray diffraction (XRD) patterns were recorded using a Bruker D8 Advance diffractometer with Cu Kα radiation. The morphology and elemental composition were examined by field-emission scanning electron microscopy (FESEM/EDS, FEI Nova NanoSEM 450) and high-resolution transmission electron microscopy (HRTEM, JEOL JEM-2010). UV–vis diffuse reflectance spectra were collected using a Cary 5000 UV–vis–NIR spectrophotometer (Agilent Technologies). The chemical states of the elements were analyzed by X-ray photoelectron spectroscopy (XPS, PHI 5000 VersaProbe II). Raman spectra were recorded using a Renishaw Raman spectrometer. The specific surface area was determined using a Micromeritics 3Flex analyzer. Solid-state photoluminescence (PL) spectra were recorded using a Horiba Fluorolog-3 spectrofluorometer. Photoelectrochemical measurements were carried out using a Biologic SP-150 electrochemical workstation in a conventional three-electrode system. Inductively coupled plasma mass spectrometry (ICP–MS) analysis was performed using a Thermo Scientific iCAP Q instrument.

### 2.6 Photocatalytic Performance Evaluation

The photocatalytic activity of the synthesized catalysts was assessed through the reduction of Cr (VI) using potassium dichromate (K_2_Cr_2_O_7_) as the Cr (VI) source at pH 3.5. The reactions were performed under visible-light irradiation provided by a 250 W tungsten lamp (λ ≥ 420 nm). A 15% aqueous NaNO_2_ solution was used as an optical filter to remove wavelengths below 420 nm. The distance between the light source and the reaction solution was maintained at 25 cm, and the light intensity at the reaction position was measured to be 0.0048 W cm^–2^. In a typical experiment, 20 mg of catalyst was dispersed in 40 mL of Cr (VI) solution (40 mg L^–1^) and magnetically stirred in the dark for 60 min to establish adsorption–desorption equilibrium. Subsequently, the suspension was irradiated for 60 min, and 1 mL aliquots were withdrawn at regular intervals to monitor the reaction progress. The reaction temperature was maintained at room temperature using a circulating water-cooling system. The reduction of Cr (VI) to Cr (III) was determined by the diphenylcarbazide (DPC) colorimetric method, with absorbance measured at 545 nm.^34^ For comparison, tetracycline (TC, 50 mg L^–1^) was employed as a model pollutant for photocatalytic degradation under the same experimental conditions, except that the reaction was carried out at neutral pH.

The degradation percentages for all experiments were calculated using the expression:

1 $$\mathrm{Degradation}\;(\%)=\frac{C_{0}-C_{t}}{C_{0}}\times 100\%$$

where *C*_0_ represents the initial concentration of the pollutant and *C*_t_ denotes the concentration at a given reaction time.

## 3 Results and Discussion

### 3.1 PXRD of Synthesized Materials

The crystal phases and structural properties of the synthesized heterostructured BWO/Ag/CuS heterostructures were examined by X-ray diffraction (XRD), as shown in Figure 1a. The presence of sharp and intense diffraction peaks indicates the formation of highly crystalline materials. The prominent diffraction peaks observed at 28.3, 32.8, 32.9, 47.0, 47.1, 55.8, 56.0, and 58.6 can be precisely attributed to the crystal planes (1 3 1), (2 0 0), (0 0 2), (2 6 0), (2 0 2), (3 3 1), (1 3 3), and (2 6 2) of orthorhombic BWO (JCPDS card no-39–0256).^35^ In addition, the diffraction pattern of CuS matches that of hexagonal covellite CuS (JCPDS card No. 78–2121).^24^ Peaks at 2θ angles of 27.7, 29.3, 31.8, 32.9, 38.8, 48.0, 52.7, and 59.3 are identified and correspond to the (1 0 1), (1 0 2), (1 0 3), (0 0 6), (1 0 5), (1 1 0), (1 0 8), and (1 1 6) planes, respectively. The PXRD pattern of the BWO/Ag/CuS composite closely resembles that of bare BWO, indicating an orthorhombic BWO structure. The absence of distinct peaks for CuS and Ag can be attributed to their low loading, small particle size, and good dispersion within the composite. The enlarged patterns shown in Figure 1b provide a clearer view of the peak positions. A slight shift of the diffraction peaks toward lower 2θ values is observed for the BWO/Ag/CuS sample compared to BWO. This shift suggests lattice expansion, which can be related to interfacial interactions between the components, leading to lattice strain within the BWO structure.^36,37^ Raman spectra further corroborate the phase composition of BWO, BWO/Ag, BWO/Ag/CuS, and CuS (Figure 1c,d). The spectra exhibit prominent bands at 795 and 828 cm^–1^, corresponding to the symmetric and antisymmetric stretching vibrations of terminal O–W–O groups. Bands observed in the 700–740 cm^–1^, region are attributed to the asymmetric stretching of WO_6_ octahedra.^38^ Bands at 262, 285, and 309 cm^–1^ are attributed to O–W–O bending vibrations, while the mode at 418 cm^–1^ corresponds to equatorial bending. Low-frequency bands around 143–154 cm^–1^ arise from Bi–O lattice vibrations.^39^ Upon Ag incorporation, a slight red shift and peak broadening of the O–W–O stretching modes are observed, indicating interaction with the BWO lattice.^40^ In addition, the characteristic band near 470 cm^–1^, assigned to S–S stretching vibration as evident from the CuS spectrum, confirms the incorporation of CuS into the composite.^41^ These variations in band position and shape reflect structural interaction among the constituent phases.

**Figure 1:**
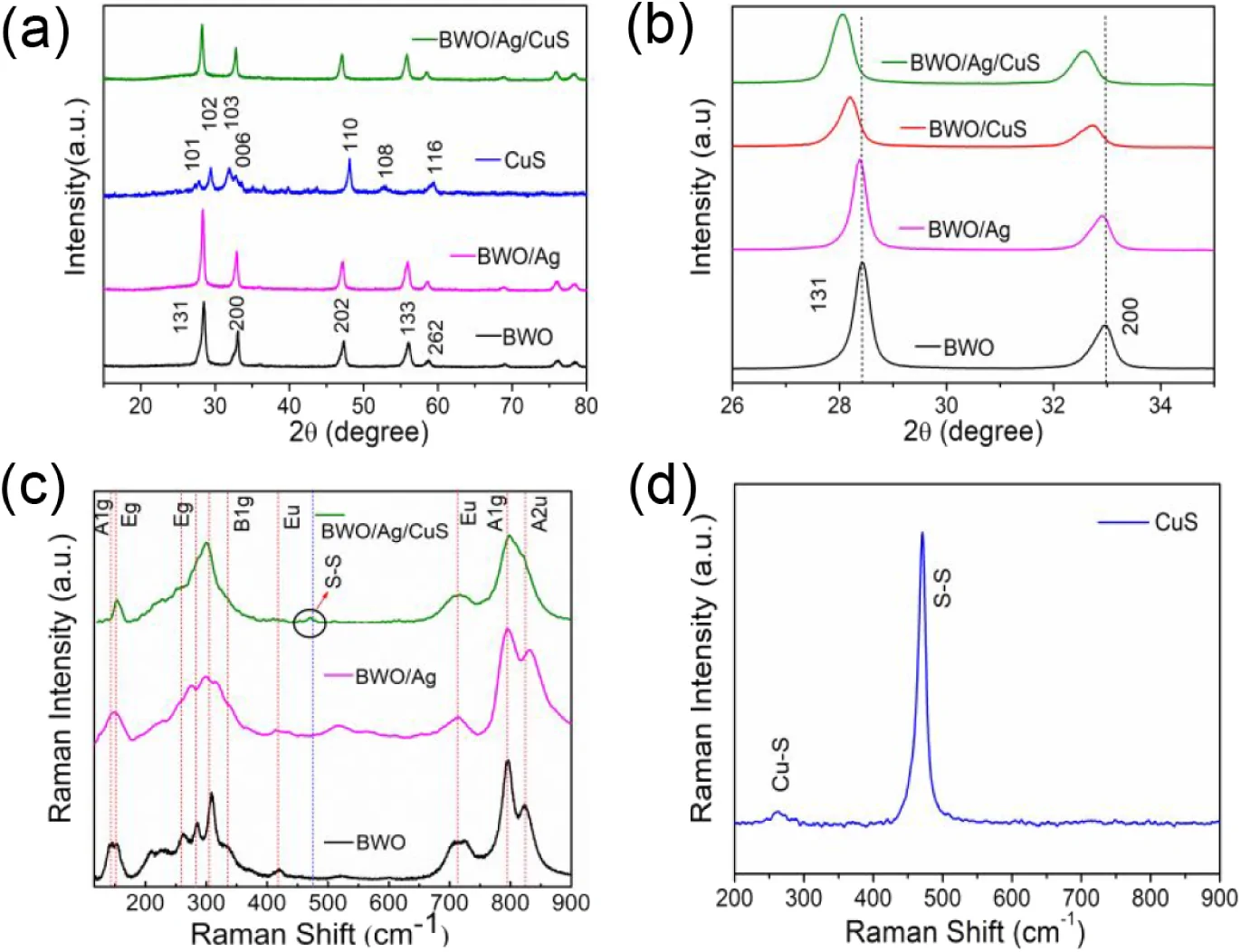
(a) PXRD patterns of the prepared BWO, BWO/Ag, CuS, and BWO/Ag/CuS nanocomposites. (b) enlarged PXRD profiles of BWO-based samples; (c) Raman spectra of BWO, BWO/Ag, and BWO/Ag/CuS nanocomposites; and (d) Raman spectra of pristine CuS.

### 3.2 Morphological Analysis

The morphology of the synthesized BWO and its composites was analyzed using FESEM. The pristine BWO exhibited a hierarchical flower-like microsphere with an average diameter of 2–4 μm, composed of interconnected nanoplates^24^ (Figure 2a). Pristine CuS particles also displayed a micro flower-like morphology (Figure 2b). The binary BWO/Ag nanocomposite retained a morphology similar to pure BWO, with cuboidal agglomerated Ag nanoparticles (70–100 nm) deposited on the BWO nanoplates (Figure S1). In the ternary BWO/Ag/CuS composite, the CuS micro flowers were anchored onto the BWO nanoplates through Ag nanoparticles, resulting in a rougher texture than the pristine BWO flowers (Figure 2c). The FESEM–EDX spectrum of the BWO/Ag/CuS composite shows the presence of Bi, W, O, Ag, Cu, and S elements without any detectable impurity signals (Figure S2). Elemental mapping further reveals a uniform distribution of these elements across the composite surface, indicating effective incorporation of Ag and CuS within the BWO matrix (Figure S3). TEM images further demonstrate the morphology of the synthesized BWO/Ag/CuS ternary composite (Figure 2d,e), revealing slight particle agglomeration with nanoparticle sizes ranging from 100 to 300 nm. The HRTEM image confirms the presence of BWO, Ag, and CuS in the composite by analyzing the lattice d-spacing (Figure 2f). BWO shows lattice fringes corresponding to the (133) planes with a d-spacing of 0.164 nm.^42^ CuS exhibits distinct fringes associated with the (102) planes at a d-spacing of 0.304 nm.^43^ In addition, cubic Ag is identified by the (002) planes with a d-spacing of 0.23 nm.^44^ These observations collectively confirm the successful formation of the BWO/Ag/CuS composite.

**Figure 2:**
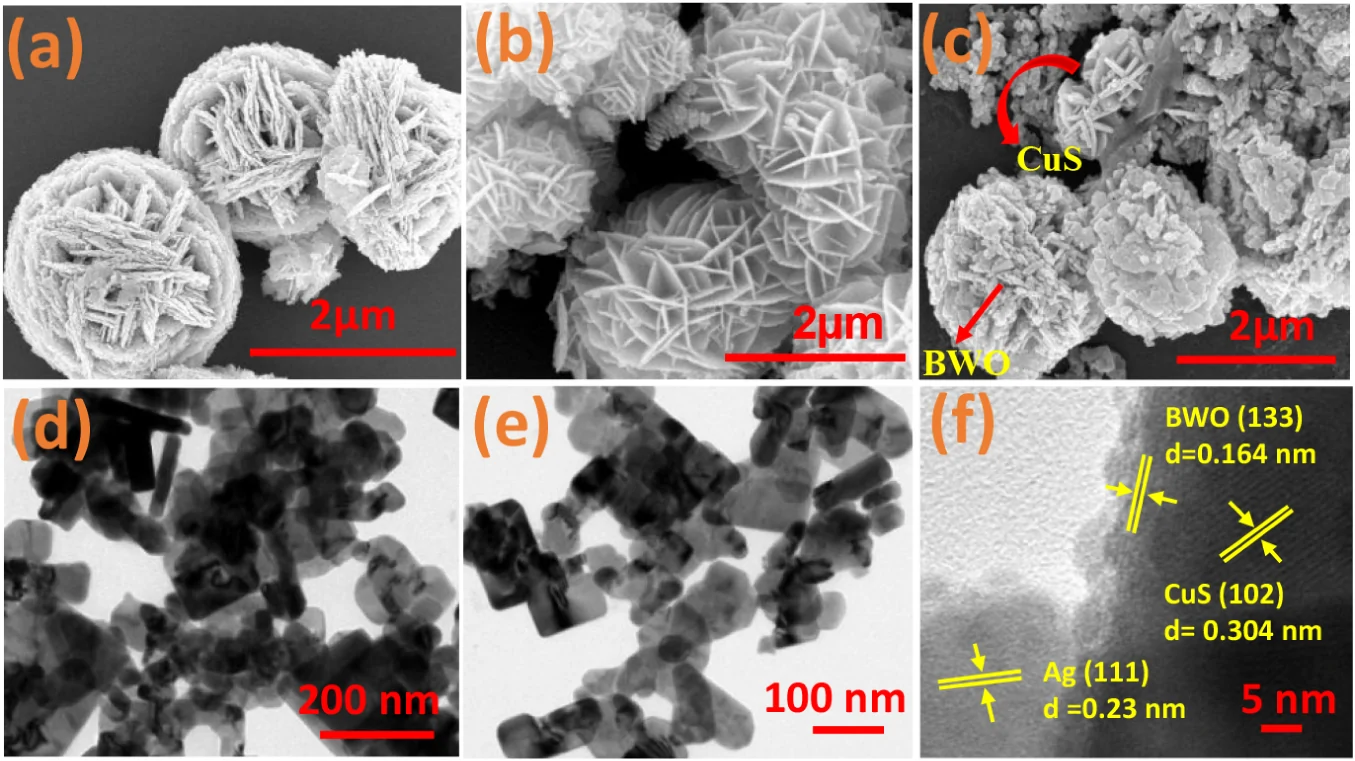
FESEM images of (a) BWO, (b) BWO/Ag, and (c) BWO/Ag/CuS, (d) TEM image of BWO/Ag/CuS nanocomposites, and (e, f) HRTEM image of the synthesized BWO/Ag/CuS.

### 3.3 Chemical Composition and Specific Surface Area Studies

X-ray photoelectron spectroscopy (XPS) was employed to investigate the surface composition and electronic structure of the BWO/Ag/CuS ternary nanocomposite. The survey spectrum (Figure 3a) showed Bi, W, O, Cu, S, and Ag and a 284.6 eV C peak, likely from hydrocarbon contamination.^45^ The high-resolution spectra of Bi 4f, W 4f, Cu 2p, O 1s, and Ag 3d for the BWO/Ag/CuS composite are shown in Figure 3b-f. In the Bi 4f spectrum (Figure 3b), two characteristic peaks located at 159.0 and 164.37 eV correspond to Bi 4f_7/2_ and Bi 4f_5/2_, respectively, with a spin–orbit splitting of approximately 5.3 eV, confirming the presence of Bi^3+^ species.^46^ Additional weak peaks at 157.2 and 162.5 eV are attributed to metallic Bi species.^47^ The W 4f spectrum (Figure 3c) shows peaks at 35.2 and 37.4 eV assigned to W^6+^ (W 4f_5/2_ and W 4f_7/2_). Additional peaks at 34.4 and 36.6 eV indicate W^5+^ species related to oxygen vacancies.^48,49^ Compared with pristine BWO the ternary composite exhibits a slight negative shift of about 0.2 eV which suggests changes in electron density due to defect formation.^50^ A weak signal near 41.9 eV is attributed to the W 5p_3/2_ orbital.^51^ High-resolution O 1s spectra with primary peaks at 529.3 and 530.7 eV corresponding to lattice oxygen species within the layer-structured Bi (Bi–O, W–O) system (Figure 3d).^52^ Two prominent peaks at 368 and 374 eV can be attributed to Ag 3d_5/2_ and Ag 3d_3/2_, respectively (Figure 3e). Peak fitting shows that the primary binding energy peaks at 367.1 and 373.1 eV are associated with Ag^+^. In contrast, the weaker peaks at 368.1 and 374.1 eV indicate the presence of metallic Ag^0^.^53,54^ In Figure 3f, Cu 2p spectra show distinct peaks for Cu 2p_3/2_ and Cu 2p_1/2_ at binding energies of 932.2 and 952.1 eV, respectively, along with a Cu^2+^ shake-up peak at 942 eV.^46^ High-resolution spectra were obtained for the S 2s region to confirm the presence of S^2–^ ions since the Bi 4f peak obscured the S 2p area. In Figure S4, the broad peak at 225.7 eV indicates the presence of sulfide ions within the CuS component.^55^ The slight positive shifts (≈0.1–0.2 eV) of the Bi 4f, W 4f, and O 1s peaks relative to pristine BWO indicate a decrease in electron density around the Bi_2_WO_6_ framework (Figure S5). Such shifts suggest interfacial charge redistribution and Fermi-level equilibration among BWO, Ag, and CuS, evidencing strong electronic coupling within the heterostructure. The resulting electronic interaction may facilitate carrier migration across the heterointerface and contribute to improved charge-separation efficiency.

**Figure 3:**
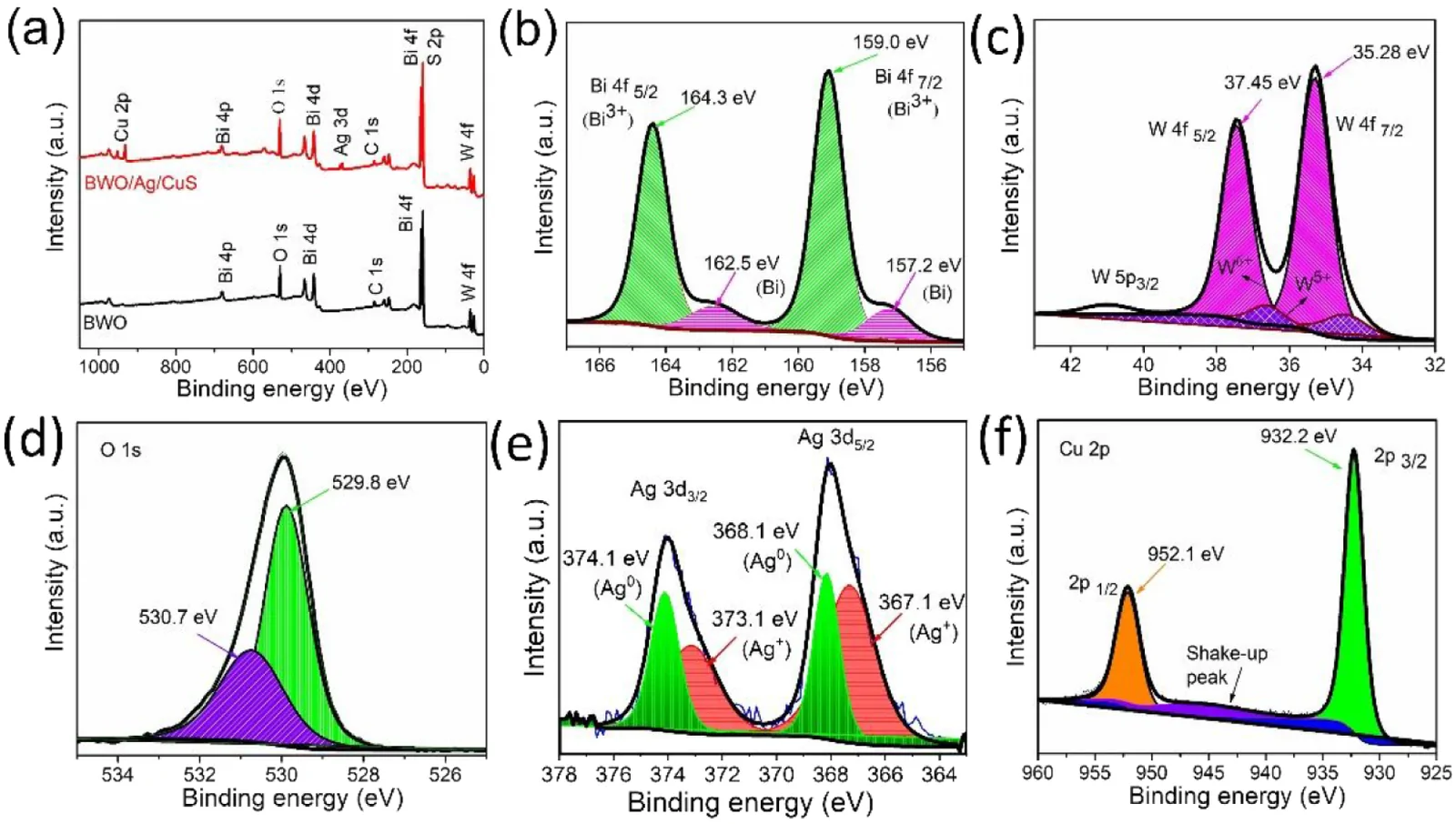
High-resolution XPS spectra of BWO/Ag/CuS nanocomposites (a) survey scan, (b) Bi 4f, (c) W 4f, (d) O 1s, (e) Ag 3d, and (f) Cu 2p.

Furthermore, the N_2_ adsorption-desorption analysis was conducted on both BWO and BWO/Ag/CuS nanocomposite (Figure S6). The BWO/Ag/CuS nanocomposite showed increased specific surface area compared to BWO. Results indicated specific surface areas of 13.05 m^2^ g^–1^ for BWO and 15.5 m^2^ g^–1^ for BWO/Ag/CuS, with pore volumes of 0.014 cm^3^ g^–1^ and 0.017 cm^3^ g^–1^. The increase in surface area can be related to the deposition of Ag and CuS nanoparticles on the BWO surface. This structural modification is expected to create additional exposed active sites and improve the interaction between the catalyst and reactant molecules, which may contribute to enhanced photocatalytic performance.

### 3.4 Optical Absorption and Photo-Electronic Properties

The optical behavior of synthesized BWO, CuS, BWO/Ag, BWO/CuS, and BWO/Ag/CuS samples were analyzed using UV-Vis diffuse reflectance spectroscopy (DRS) and photoluminescence (PL). As shown in Figure 4a, BWO has an absorption edge starting at around 450 nm, while CuS exhibits extensive absorption across the visible spectrum. The incorporation of Ag nanoparticles into BWO improves visible-light absorption and produces an additional band around 510 nm, which is associated with the surface plasmon resonance (SPR) effect of Ag nanoparticles.^31^ The further introduction of CuS into the BWO/Ag system extends the absorption range from the UV–visible region to the near-infrared region, enabling the ternary composite to utilize a wider portion of the solar spectrum. Band gap energies calculated from the Tauc plots (Figure 4b) are 2.60 eV for BWO, 2.29 eV for BWO/CuS, and 2.16 eV for BWO/Ag/CuS, indicating improved light-harvesting capability in the composite materials. Photoluminescence spectra were recorded with an excitation wavelength of 330 nm to evaluate charge carrier recombination. All samples display an emission peak near 468 nm, while pristine BWO shows the highest PL intensity (Figure S7), indicating rapid recombination of photogenerated carriers. In contrast, the BWO/Ag/CuS composite exhibits the weakest PL emission, suggesting more efficient separation of electron–hole pairs due to the interaction among BWO, Ag, and CuS components.

**Figure 4:**
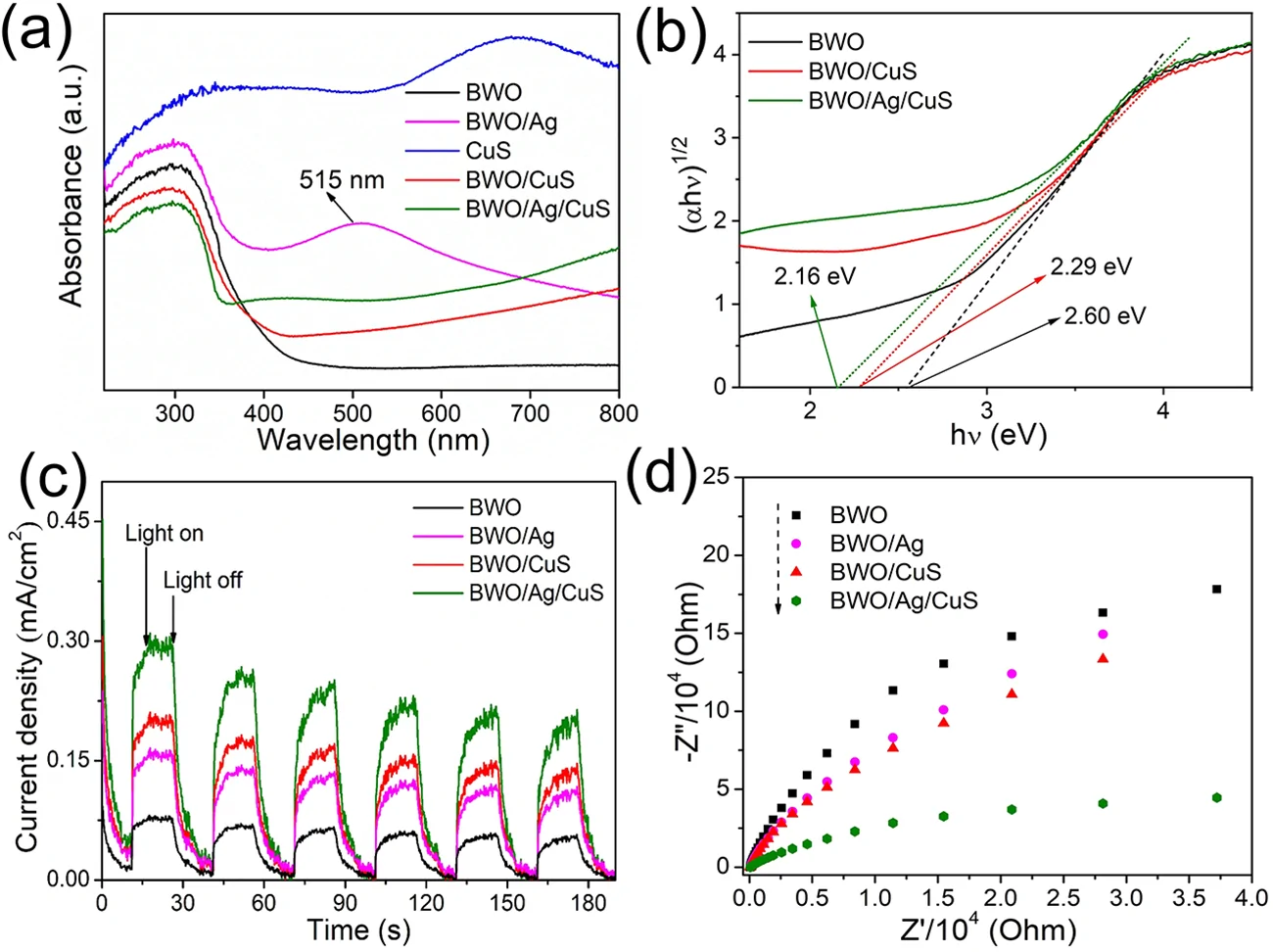
(a) UV–vis absorption spectra (DRS), (b) Tauc plots of pristine BWO, BWO/CuS, and BWO/Ag/CuS, (c) and (d) transient photocurrent response and EIS Nyquist plot of BWO, BWO/Ag, BWO/CuS, and BWO/Ag/CuS.

To further investigate the charge transfer behavior, photoelectrochemical measurements such as photocurrent response (PC) and electrochemical impedance spectroscopy (EIS) were carried out. As shown in Figure 4c, the BWO/Ag/CuS composite exhibits the highest photocurrent density among all samples, indicating more efficient separation and migration of photogenerated charge carriers. All the prepared samples displayed stable and repeatable photocurrent profiles during successive light irradiation cycles, reflecting excellent photostability. The EIS results (Figure 4d) show that the BWO/Ag/CuS composite possesses the smallest semicircle radius in the Nyquist plot, which corresponds to the lowest charge transfer resistance. The resistance follows the order BWO > BWO/Ag > BWO/CuS > BWO/Ag/CuS, confirming that the ternary composite promotes faster interfacial charge transfer and suppresses electron–hole recombination.

### 3.5 Photocatalytic Degradation of TC

The photocatalytic activity of the synthesized materials was evaluated using tetracycline (TC) as a model pollutant under visible-light irradiation. Before illumination, all suspensions were stirred in the dark for 60 min to attain adsorption–desorption equilibrium, followed by 60 min of photocatalytic degradation. The effects of Ag and CuS loading, together with other parameters influencing TC removal, were systematically examined.

#### 3.5.1 Impact of Ag and CuS Contents

The effects of Ag and CuS loading on the photocatalytic degradation of TC were investigated to identify the optimum composition of the ternary heterostructure. As shown in Figure S8a, the photocatalytic activity of BWO/Ag composites initially increased with increasing Ag content and reached a maximum at 4 wt % Ag. The optimized BWO/4%Ag sample achieved 63% degradation within 60 min, compared to 50% for pristine BWO. The enhanced activity can be attributed to improved visible-light utilization and charge separation induced by the incorporation of Ag nanoparticles. However, a further increase in Ag content resulted in a gradual decline in photocatalytic activity, which may be due to the formation of excessive Ag domains that promote charge recombination and partially block active surface sites. To further enhance the photocatalytic performance, CuS was introduced into the optimized BWO/4%Ag composite to construct a ternary BWO/Ag/CuS heterostructure. As shown in Figure S8b, the degradation efficiency increased with increasing CuS content and reached a maximum at 10 wt %, followed by a decrease at 15 wt %. The improved activity at lower CuS loading is attributed to the formation of an efficient heterojunction that facilitates charge separation and transfer. However, at higher CuS loading, the formation of CuS–CuS homojunctions promotes charge carrier recombination, leading to a decrease in photocatalytic efficiency. These results indicate that an optimal loading of Ag and CuS is essential to achieve efficient charge separation and improved photocatalytic activity. For comparison, the photocatalytic degradation of TC over BWO, CuS, BWO/Ag, BWO/CuS, and BWO/Ag/CuS was evaluated under identical conditions (Figure 5a). At first, the prepared catalyst was mixed with TC solution and stirred in the dark for 60 min to reach adsorption equilibrium. The ternary BWO/Ag/CuS composite showed the highest adsorption rate, likely due to its larger surface area, and achieved 92% TC degradation within 60 min irradiation. Based on the UV–Vis calibration curve (Figure S8c), the residual TC concentration decreased from an initial 50 mg L^–1^ to approximately 3.5 mg L^–1^ after 60 min of photocatalytic treatment. As shown in Figure 5b, the characteristic absorption band of TC at 357 nm undergoes a noticeable redshift after contact with the BWO/Ag/CuS photocatalyst, followed by a progressive decrease in absorption intensity during visible-light irradiation. This peak shifting can be attributed to interactions between TC molecules and surface oxygen-vacancy sites, which modify the electronic structure of TC and enhance π-conjugation.^56^ The subsequent decrease in absorption intensity reflects the continuous degradation of TC during the photocatalytic process.

**Figure 5:**
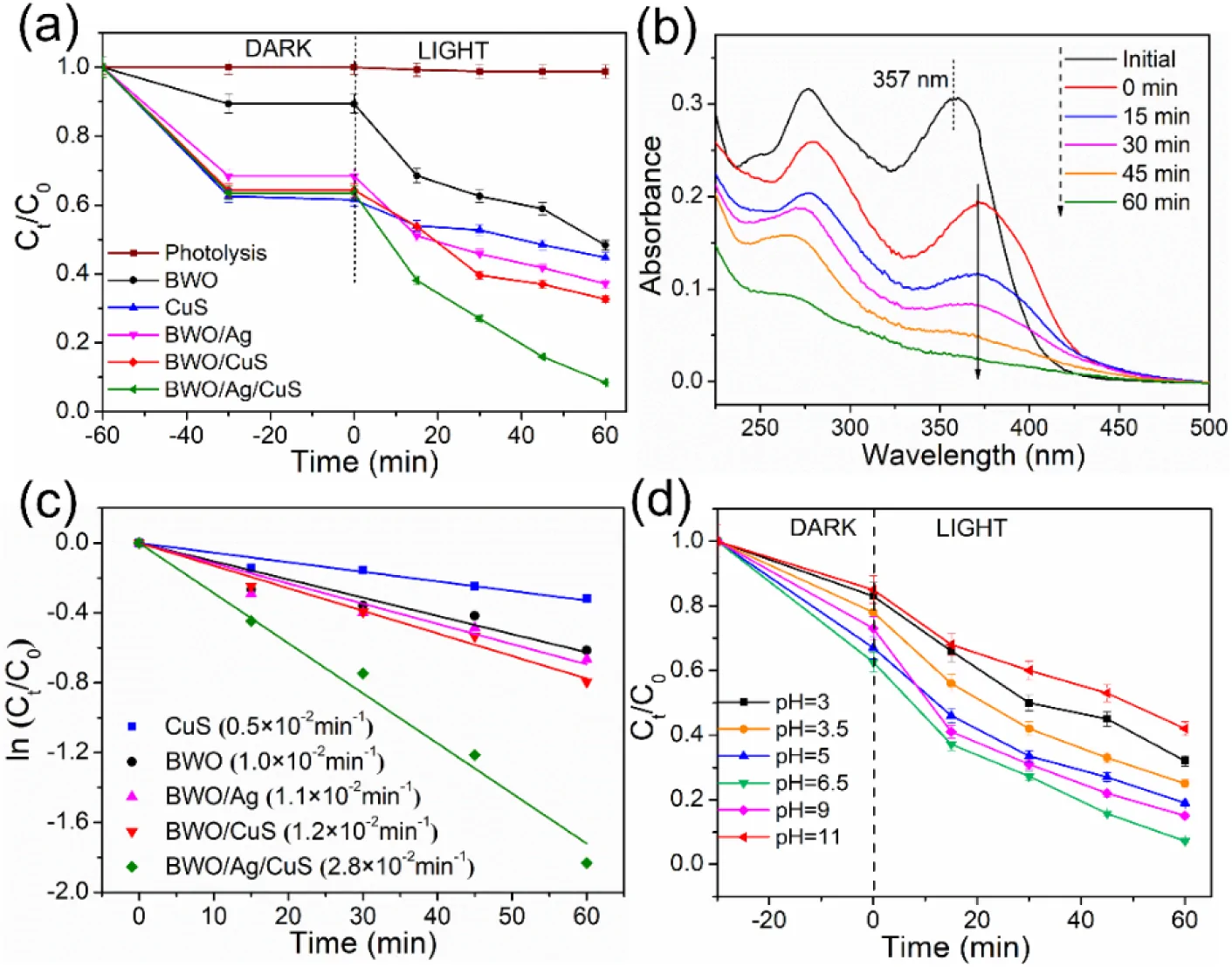
(a) Plot of (C_t_/C_0_) *vs* time for photodegradation of TC, (b) UV-Vis absorption spectra of TC at various intervals of times, (c) first-order kinetic study for TC degradation, and (d) effect of initial solution pH on the degradation of TC over of BWO/Ag/CuS nanocomposite.

The photodegradation process was studied using a pseudo-first-order kinetic model to determine reaction kinetics. The reaction rate constant (k, min^–1^) can be calculated using the formula.^57^

2 $$\ln\;\left(C_{t}/C_{0}\right)=kt$$

*C*_t_ and *C*_0_ are the TC concentrations at different time intervals and 0 min, respectively (Figure 5c), showing the prepared catalyst’s apparent rate constant (k). The pristine BWO and CuS exhibit relatively low k-values of 0.009 min^–1^ and 0.005 min^–1^, respectively. In contrast, ternary BWO/Ag/CuS nanocomposites have higher k values than other materials evaluated, with the highest value being 0.0336 min^–1^ under the same condition. The k-values of the ternary system are 6.72 times and 3.73 times greater than those of CuS and BWO, respectively. To distinguish the effect of heterostructure formation from simple component mixing, a comparison with a mechanically mixed (BWO + Ag + CuS) sample was performed (Figure S8d). The synthesized BWO/Ag/CuS heterostructure exhibited significantly higher photocatalytic activity than the physical mixture. This enhancement results from the well-established heterointerface formed during synthesis, which promotes efficient interfacial charge transfer and suppresses charge-carrier recombination.

#### 3.5.2 Effects of Operational Factors on Photocatalytic TC Degradation

##### 3.5.2.1 Impact of Solution pH on TC Degradation

The pH of the reaction medium significantly influences photocatalytic TC degradation by affecting both TC speciation and the surface charge of the photocatalyst. As shown in Figure 5d, the degradation efficiency strongly depended on solution pH, with the highest activity observed at near-neutral conditions. Zeta-potential measurements (Figure S9a) revealed a pHzpc of approximately 4.3, indicating a positively charged surface below this value and a negatively charged surface above it. TC predominantly exists as the cationic species (H_3_A^+^) at pH < 3.3, the zwitterionic species (H_2_A) between pH 3.3 and 7.7, and the anionic species (HA^–^ and A^2–^) at pH > 7.7.^58^ At near-neutral pH, where TC mainly exists as H_2_A, favorable interactions between TC molecules and the catalyst surface promote adsorption and degradation. In contrast, under strongly alkaline conditions (pH 11), both the catalyst surface and TC molecules are negatively charged, leading to electrostatic repulsion and reduced degradation efficiency. In strongly acidic media, excess H^+^ ions consume photogenerated electrons and hinder ^•^O_2_^–^ generation, resulting in lower photocatalytic activity.^59^ These results indicate that near-neutral pH provides the most favorable environment for TC degradation. Therefore, the reduced activity observed under strongly acidic and alkaline conditions is primarily associated with pH-dependent TC speciation and surface-charge interactions.

##### 3.5.2.2 Impact of Initial TC Concentration on TC Degradation

The initial concentration of TC also affects the process of degradation. In Figure S9b shows that the degradation efficiency decreased from 92% to 49% with an increase in the concentration of the TC between 50 and 80 mg L^–1^. This reduction can be explained by the limited penetration of light and the increased competition among TC molecules for the available active sites on the catalyst surface.^56^ Therefore, an initial TC concentration of 50 mg L^–1^ was selected as the optimal condition for subsequent photocatalytic experiments. Furthermore, the kinetic behavior of TC degradation at a lower concentration (20 mg L^–1^) was analyzed for different synthesized samples (Figure S9c). The ternary BWO/Ag/CuS composite shows a kinetic rate constant of 0.0635 min^–1^, which is approximately 9.33 times higher than that of pristine BWO, highlighting the superior photocatalytic performance of the ternary system.

##### 3.5.2.3 Impact of Inorganic Ions and Humic Acid on TC Degradation

The influence of common inorganic ions and natural organic matter (NOM), represented by humic acid (HA), on TC degradation was evaluated (Figure S9d). HA produced the strongest inhibition, reducing the degradation efficiency to 45%. This decrease mainly results from competitive adsorption on the catalyst surface, scavenging of reactive oxygen species, and light-shielding effects. Among the inorganic ions, Cl^^–^^ and CO_3_^2^–^^ caused the greatest decrease in photocatalytic activity, lowering the degradation efficiency to 68% and 72%, respectively. The inhibition by Cl^^–^^ results from competitive adsorption and its reaction with photogenerated holes and ^•^OH radicals to form less reactive chlorine radical species (^•^ClOH^–^ and ^•^Cl), which reduces the availability of reactive species for TC degradation. CO_3_^2 –^ reacts with the dominant ^•^O_2_^^–^^ radicals to produce less reactive CO_3_^•^–^^ radicals, leading to a further decrease in degradation efficiency. Similarly, SO_4_^2^–^^ also reduced the photocatalytic activity by reacting with photogenerated holes to form SO_4_^•^–^^ radicals, which have lower oxidation activity toward TC than ^•^OH radicals.^59,60^ In contrast, NO_3_^^–^^ had only a minor influence, and the degradation efficiency remained at 87% because of its relatively low reactivity toward the reactive species involved in the photocatalytic process. These results show that the BWO/Ag/CuS photocatalyst maintains satisfactory activity in the presence of common water constituents.

##### 3.5.2.4 Influence of Real Water Matrices on TC Degradation

The practical applicability of the photocatalyst was further assessed using tap water and wastewater as reaction media (Figure S9e). Compared with deionized water, the degradation efficiency decreased slightly to 85% in tap water, likely due to the presence of turbidity and low concentrations of dissolved ions. A more noticeable decline was observed in wastewater, where the degradation efficiency reached 72%. This reduction can be attributed to the higher levels of inorganic ions, organic matter, and suspended solids, which interfere with pollutant adsorption and light transmission. However, the photocatalyst maintained appreciable activity in both water matrices, demonstrating its potential for application in complex water environments.

#### 3.5.3 Photocatalytic Degradation Pathway of TC and Phytotoxicity Assessment

To clarify the degradation mechanism of TC over the BWO/Ag/CuS photocatalyst, LC–MS analysis was performed to identify the major photodegradation intermediates (Figure 6). The chromatograms shown in Figure S10 compare the reaction mixtures collected at 0, 30, and 60 min. The characteristic TC peak observed at a retention time of 0.80 min decreased significantly with increasing irradiation time, indicating progressive degradation of the parent molecule. The corresponding mass spectra (Figure S11) revealed several intermediate species formed during the reaction. The peak at m/z = 445 detected at 0 min corresponds to the parent TC molecule. Previous studies have reported that TC is highly susceptible to attack by reactive oxygen species owing to the presence of electron-rich functional groups, including the C=C bond, phenolic hydroxyl groups, and amino substituents. In pathway I, hydroxyl radical attack on the Ca-Cb bond leads to the formation of the hydroxylated intermediate P1 (m/z = 461), while subsequent hydroxylation generates P2 (m/z = 477), which is consistent with previously reported LC–MS analyses of TC degradation.^61–63^ As the reaction proceeds, P2 undergoes successive oxidation, ring opening, demethylation, and fragmentation reactions to produce lower-molecular-weight species such as P3 (m/z = 214), as reported in earlier photocatalytic studies.^62,64^ In pathway II, TC is initially attacked by reactive species and subsequently undergoes oxidation and ring-opening reactions through intermediate P4, ultimately yielding P5 (m/z = 228), which agrees well with degradation products identified in previous reports.^53,62^ Continued oxidation of these intermediates results in the formation of smaller organic fragments, which are eventually mineralized into CO_2_, H_2_O, and inorganic nitrogen-containing species.^60,65–67^

**Figure 6:**
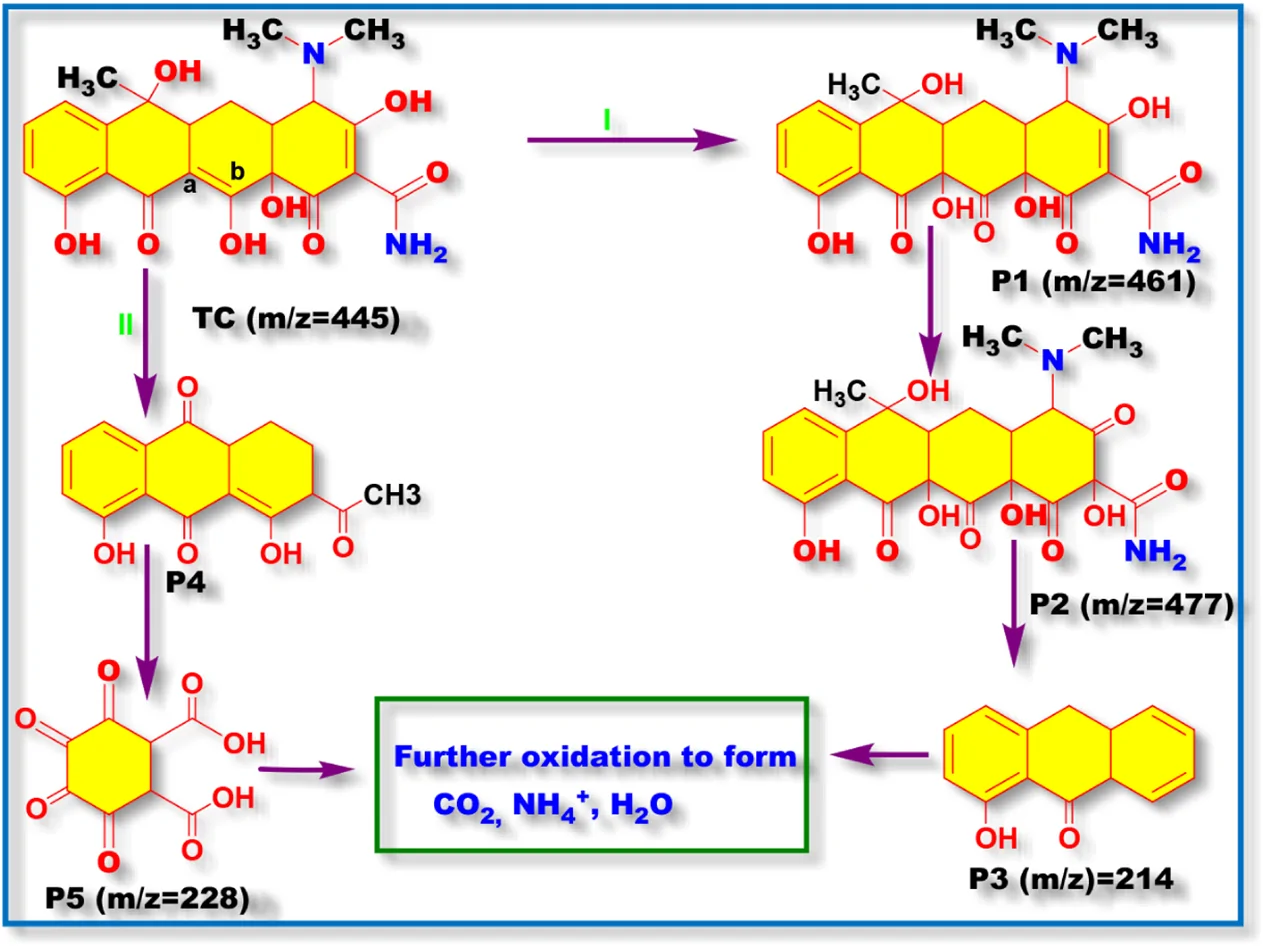
Possible photodegradation pathways and intermediate products of TC.

To ensure the environmental safety of the treated water, ecotoxicity was evaluated using Vigna radiata (mung bean) germination, as illustrated in Figure 7. Germination tests were conducted at ambient temperature using distilled water (a1–a3), photocatalytically treated TC solution (b1–b3), photocatalyst dispersion (10 mg/50 mL) (c1–c3), and untreated TC solution (50 mg/L) (d1–d3). After 10 days, the hypocotyl lengths in distilled water, treated TC solution, and catalyst dispersion were approximately 12.5, 11.0, and 10.5 cm, respectively. The catalyst-dispersion group exhibited normal seed germination and root development, while severe growth inhibition and lesion formation were observed in the untreated TC solution. In contrast, the treated TC solution showed substantially improved root growth, indicating a significant reduction in TC toxicity after photocatalytic treatment. This behavior is likely related to the lower residual tetracycline concentration, which promotes the accumulation of reactive oxygen species (ROS) in the seeds. Consequently, the enhanced oxidative status of the roots accelerates seed germination and growth.These findings demonstrate that the BWO/Ag/CuS photocatalyst-treated solution is nontoxic to mung seed growth and can be safely used for agricultural irrigation.

**Figure 7:**
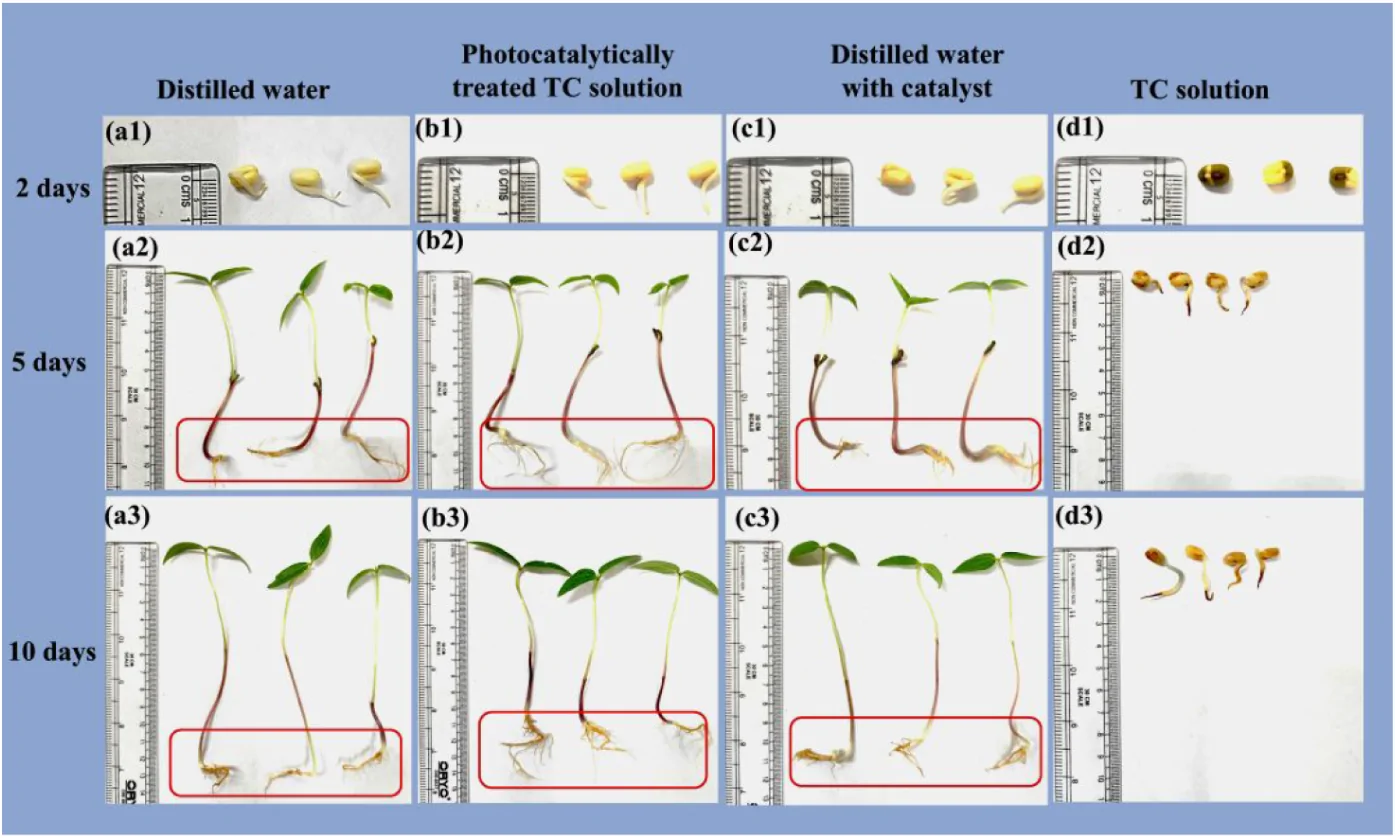
Toxicity investigation for the growth of vigina radiata: (a1-a3) distilled water, (b1-b3) photocatalyzed TC solution, (c1-c3) distilled water with catalyst, and (d1-d3) TC solution treated water.

### 3.6 Photocatalytic Reduction of Cr (VI)

The photocatalytic activity of the prepared materials was further evaluated to reduce toxic Cr (VI) to less harmful Cr (III) (Figure 8a). The ternary BWO/Ag/CuS nanocomposites demonstrated a significant decrease in Cr (VI) concentration, achieving a 90% reduction within 60 min. Absorption spectra (Figure 8b) confirmed effective Cr (VI) elimination. The 1,5-diphenylcarbazide (DPC) method was used for accurate Cr (VI) quantification at 540 nm, with the solution’s color change from pink to colorless, indicating successful reduction.^68^ This color change occurs because Cr (III) cannot form a complex with DPC due to its stability and inertness, while the highly oxidizing Cr (VI) forms a pink octahedral complex. The photoreduction followed a pseudo-first-order kinetic model (Figure 8c), with BWO/Ag/CuS showing the highest k-value of 0.0322 min^–1^, significantly higher than the k-values for BWO (0.0039 min^–1^) and CuS (0.003 min^–1^), indicating enhanced catalytic efficiency. Further, the kinetic behavior of Cr (VI) photoreduction at a lower concentration (20 mg/L) for various prepared samples was analyzed and is presented in (Figure S12). The ternary composite exhibits a k-value of 0.0712 min^–1^, 10.17 times greater than that of pristine BWO. To investigate the influence of solution pH on the reduction of Cr (VI), experiments were conducted across a pH range of 3.5 to 8.25 (Figure 8d). pH was adjusted using 0.1 M HCl or 0.1 M NaOH, as Cr (VI) species (HCrO_4_^–^, Cr_2_O_7_^2–^, CrO_4_^2–^) are strongly affected by pH. Maximum reduction (90%) occurred at pH 3.5 due to enhanced adsorption of Cr_2_O_7_^2–^ and HCrO_4_^–^ on a more protonated surface. Conversely, at pH 8.25, reduction efficiency decreased by 17.5%, likely due to increased OH^–^ adsorption overshadowing anion adsorption (CrO_4_^2–^). Additionally, the formation of Cr (OH)_3_ precipitates blocked the active sites of the catalyst, further reducing reduction efficiency.^69^ Thermodynamically, the reduction of Cr_2_O_7_^2–^ to Cr^3+^ is more favorable than that of CrO_4_^2–^ to Cr (OH)_3_. Acidic conditions are thus advantageous, as the standard potential E° (Cr_2_O_7_^2–^ /Cr^3+^) is significantly higher (+1.36 eV vs. NHE) than E° (CrO_4_^2–^ /Cr (OH)_3_) (−0.13 eV vs. NHE).^63^

3 $$Cr_{2}{O_{7}}^{2-}+14H^{+}+6e^{-}\to 2Cr^{3+}+7H_{2}O$$

4 $$Cr{O_{4}}^{2-}+4H_{2}O+3e^{-}\to Cr{\left(OH\right)}_{3}+5OH^{-}$$

**Figure 8:**
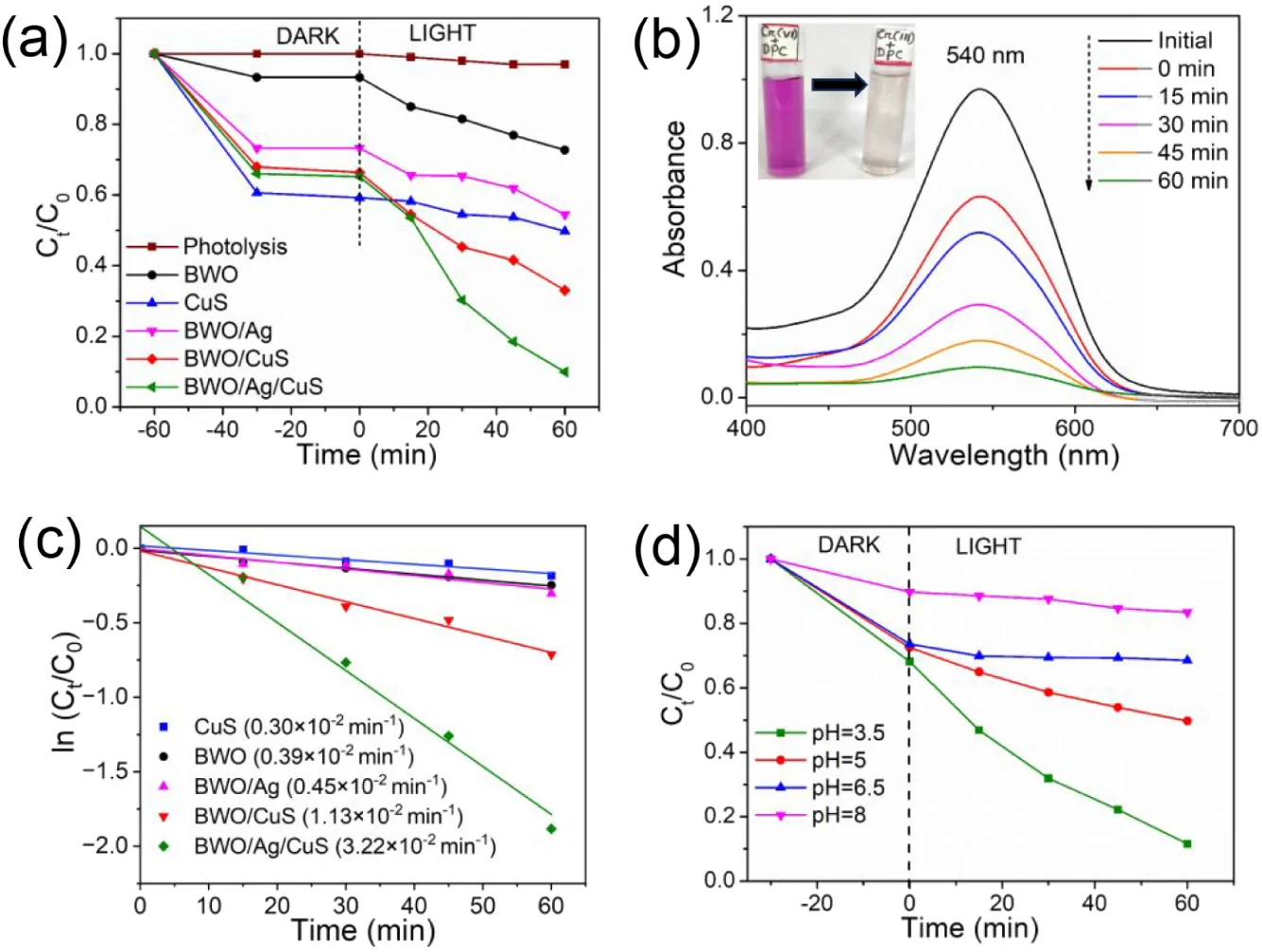
(a) Plot of (*C*_t_/*C*_0_) *vs* time for Cr (VI) photoreduction kinetics; (b) absorption spectra of Cr (VI) with BWO/Ag/CuS nanocomposite under visible light irradiation; (c) plot of ln (*C*_t_/*C*_0_) *vs* time for Cr (VI) photoreduction kinetics; and (d) impact of Initial pH on Cr (VI) reduction in the presence of BWO/Ag/CuS nanocomposite.

### 3.7 Synergistic Photocatalytic Removal of Co-Existing TC and Cr (VI)

To evaluate the interaction between the oxidation and reduction processes, photocatalytic experiments were further conducted using a mixed TC/Cr (VI) solution under visible-light irradiation at pH 3.5. For comparison, TC degradation and Cr (VI) reduction were also examined individually under identical conditions. As shown in Figure S13, the degradation efficiency of TC increased from 73% in the single-pollutant system to 80% in the mixed system, while the reduction efficiency of Cr (VI) increased from 90% to 97%. The simultaneous enhancement of both reactions indicates a synergistic effect between TC oxidation and Cr (VI) reduction. In the coupled system, TC acts as a hole-consuming species, whereas Cr (VI) serves as an electron acceptor. The concurrent consumption of photogenerated holes and electrons suppresses charge-carrier recombination and promotes charge separation, thereby enhancing both oxidation and reduction processes.^53,67^ These results further support the efficient charge-transfer characteristics of the Ag-mediated Z-scheme BWO/Ag/CuS photocatalyst.

### 3.8 Identification of Reactive Oxygen Species

Reactive oxygen species (ROS) generated during photocatalysis play an essential role in the degradation of organic pollutants. To determine the main active species involved in the reaction, radical scavenging experiments were carried out using specific quenching agents. p-Benzoquinone (BQ) was used to trap superoxide radicals (^•^O_2_^–^), EDTA-2Na served as a hole (h^+^) scavenger, and isopropanol (IPA) was employed to capture hydroxyl radicals (^•^OH).^56^ As presented in Figure 9a,b, the degradation of TC was significantly inhibited by h^+^, **^•^** OH, and ^•^O_2_^–^. The significant inhibition of TC degradation under an N_2_ atmosphere indicates the involvement of dissolved oxygen in the photocatalytic process, while the participation of ^•^O_2_^–^ was further supported by the BQ scavenging experiment. To further investigate the roles of ^•^OH and ^•^O_2_^–^ in the photocatalytic activity of the BWO/Ag/CuS nanocomposite, DMPO spin-trapping experiment was employed. Under dark conditions, no significant signals for DMPO–^•^OH or DMPO–^•^O_2_^–^ adducts were detected (Figure 9c,d). Upon light irradiation, a distinct 1:2:2:1 quartet signal assigned to DMPO–^•^OH, indicating that photoinduced holes react with adsorbed H_2_O to generate hydroxyl radicals.^14^ In contrast, when the reaction medium was changed from water to methanol, DMPO effectively formed an adduct with ^•^O_2_^–^, producing distinct signals compared to ^•^OH.^70^ The EPR analysis confirmed that the BWO/Ag/CuS nanocomposite generates both ^•^O_2_^–^ and ^•^OH under visible light irradiation, which agrees well with the radical trapping experiments. These findings demonstrate that the photocatalytic performance of pure BWO is significantly improved by incorporating a multi-heterojunction structure formed by Ag NPs, CuS, and BWO within the BWO/Ag/CuS composite.

**Figure 9:**
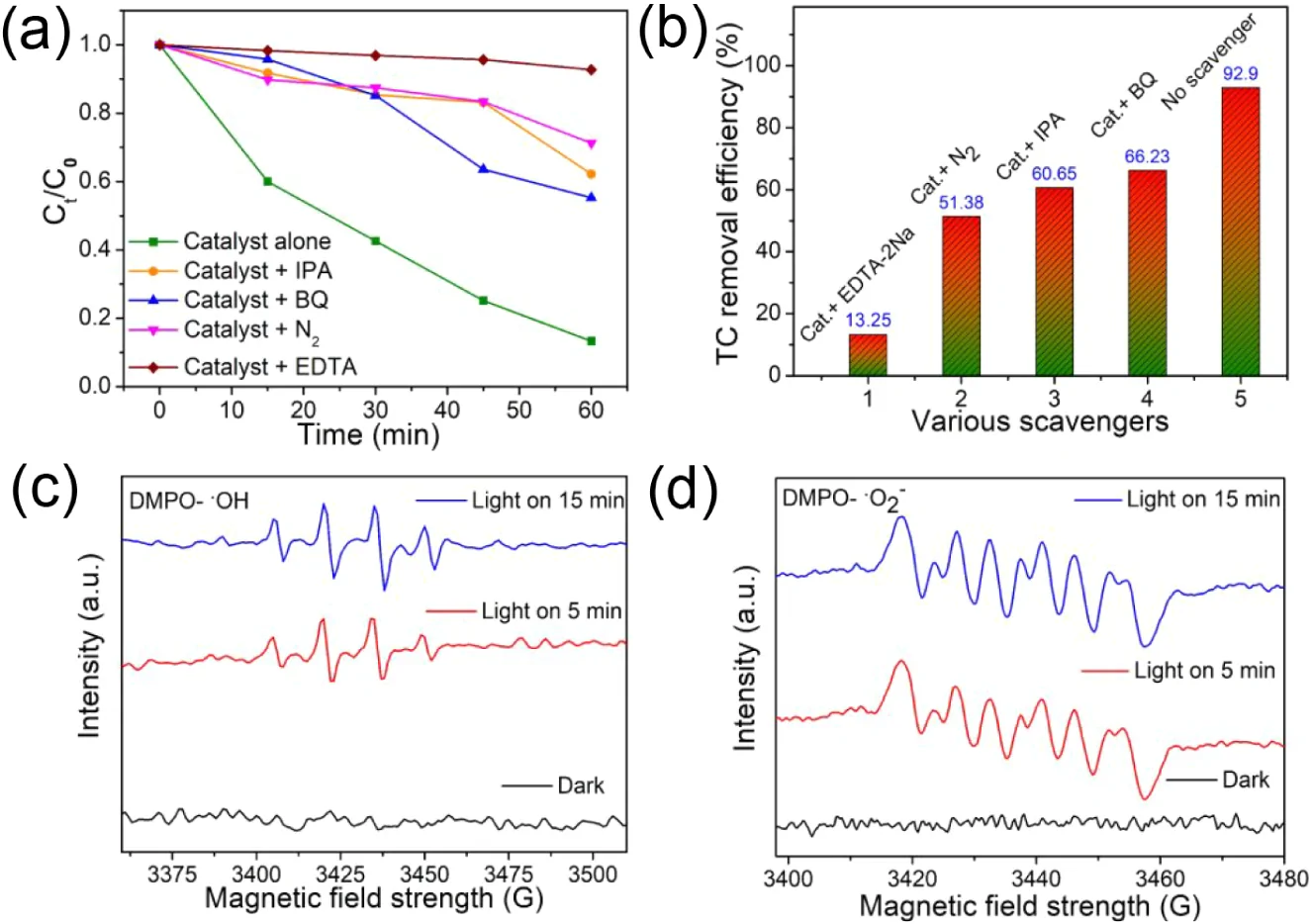
(a) Impact of various scavengers on the photodegradation of TC; (b) histogram analysis of the impact of radical scavengers on TC degradation; (c) EPR spectra of BWO/Ag/CuS in aqueous dispersion for DMPO-**^•^** OH and (d) methanol dispersion for DMPO-^•^O_2_^–^.

### 3.9 Photocatalytic Activity: Mechanisms and Modes in Binary and Ternary Nanocomposites

Based on the observation, a potential charge transfer mechanism for the BWO/Ag/CuS nanocomposite in TC and Cr (VI) removal is proposed in Scheme 2. Two typical charge transfer processes (traditional p-n heterojunction and Z-scheme) may coexist and compete in most semiconductor photocatalysts. To clarify the mechanism in the BWO/Ag/CuS system, band edge values of BWO and CuS were determined using Mott-Schottky plots (Figure S14) and UV-vis DRS spectra (Figure 4). Mott-Schottky analysis determined the pristine material’s flat band potential and semiconductor type (n- or p-type) at 1000 Hz. BWO showed a positive slope, indicating an n-type semiconductor with E_fb_ at approximately −0.37 V (vs. Ag/AgCl).^71^ In contrast, CuS showed a negative slope, suggesting a p-type semiconductor with E_fb_ at 1.0 V relative to Ag/AgCl. E_fb_ is often equivalent to the bottom of the conduction band (CB) for n-type semiconductors and the valence band (VB) for p-type semiconductors.^66^ The CB of BWO and VB of CuS photocatalyst was estimated at −0.28 and 1.39 V versus NHE, respectively, calculated using the following equation.^66^

5 $$E_{NHE,PH=7}=E_{Ag/AgCl,pH=6.6}-0.059\left(7-\mathrm{pH}\;\mathrm{of}\;\mathrm{electrolyte}\right)+0.198$$

6 Bandgap(eV)=Valenceband(eV)−conductionband(eV)

**Scheme 2:**
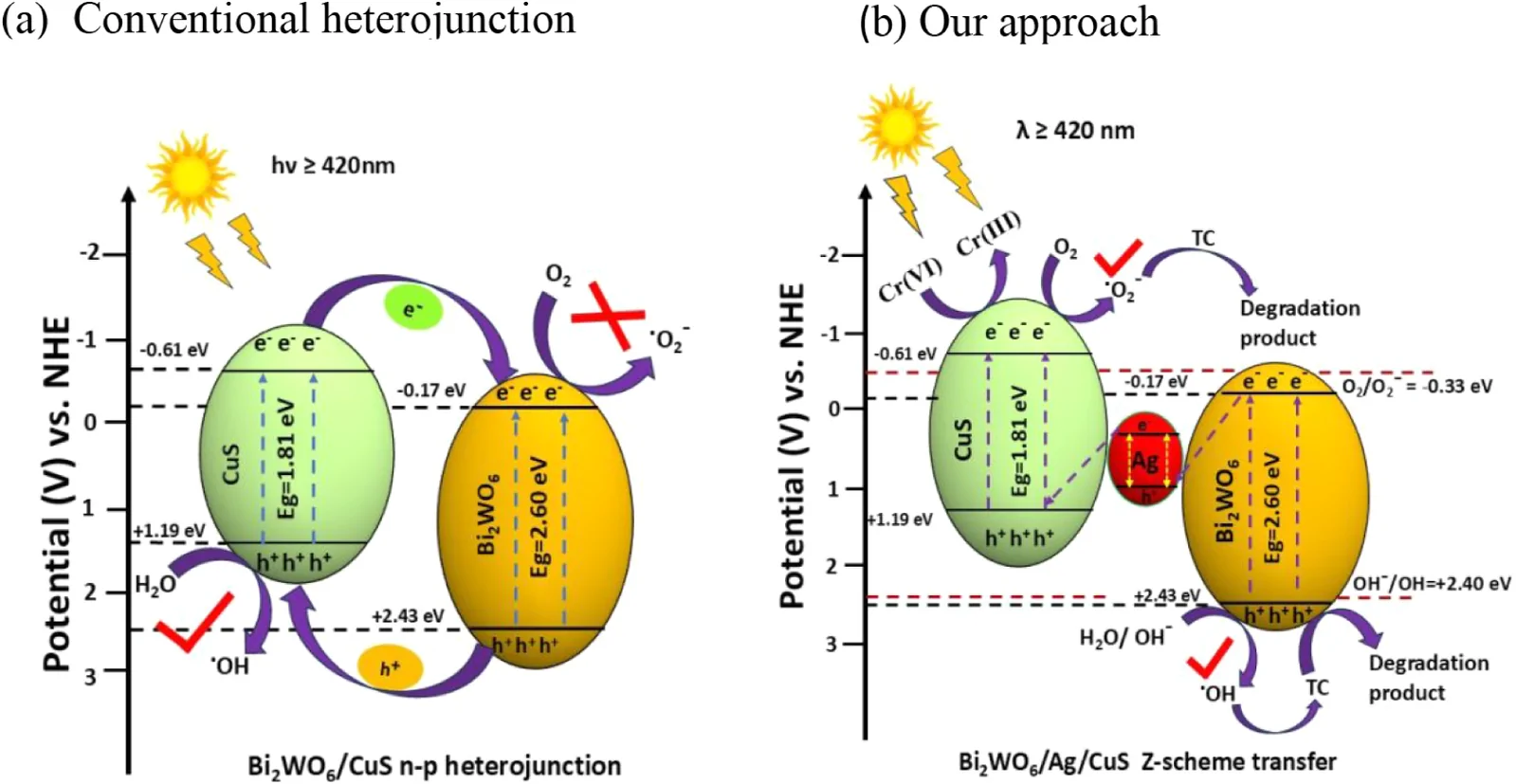
Possibe Photocatalytic Mechanism in the Binary BWO/CuS (a) and Ternary BWO/Ag/CuS (b) Nanocomposite Under Visible Light Irradiation

The band gaps of the pristine BWO and CuS as 2.60 and 1.81 eV, respectively, determined from Tauc plots. Calculated VB for BWO and CB for CuS was approximately 2.44 eV and −0.41 eV (vs. NHE), respectively, using equation 3 above.^72^ Under visible light irradiation, photogenerated electrons (e^–^) transfer from the CB of CuS to that of BWO, while holes (h^+^) migrate from the VB of BWO to CuS. As a result, electrons accumulate in the CB of BWO and holes in the VB of CuS, promoting efficient charge separation (Scheme 2a). The separated electrons cannot reduce O_2_ to ^•^O_2_^–^ because its theoretical reduction potential is −0.33 V, compared to the CB potential of BWO (−0.28 V vs NHE). In general, CB of BWO cannot generate ^•^O_2_^–^ radicals. CuS has a more negative VB potential (+1.4 V vs. NHE) than (H_2_O/^•^OH) at (+2.4 V vs NHE) and (OH^–^/**^•^** OH) at (+1.99 V vs NHE), hence the VB of CuS lacks energy, it cannot generate hydroxyl radicals.^23^ The discussion and results suggest a Z-scheme type photocatalytic mechanism for BWO/Ag/CuS catalyst (Scheme 2b). This approach balances BWO, Ag, and CuS Fermi levels before light irradiation. BWO and CuS photoexcited by visible light generate electrons and holes. Photogenerated electrons from the CB of BWO move to metallic Ag, while Ag NPs strong surface plasmon resonance (SPR) boosts the contact’s local electric field. This field permits Ag photogenerated electrons to recombine with CuS VB holes via plasmon-induced resonance energy transfer or direct electron migration. Afterward, photogenerated (e^–^) in the CB of CuS combine with dissolved O_2_ to form ^•^O_2_^–^, accumulating holes in VB of BWO degrade TC molecules by direct interaction or interactions with H_2_O or OH^–^ to produce ^•^OH radicals. In Cr (VI) reduction, photogenerated electrons directly reduce Cr (VI) to Cr (III). This electron-hole migration route conserves photogenerated electrons in the negative CB of CuS and holes in the positive VB of BWO, thereby promoting efficient charge separation while preserving their strong redox ability. In addition to its SPR effect, Ag serves as an electron mediator between BWO and CuS. The observed XPS binding-energy shifts suggest interfacial charge redistribution and strong electronic coupling among BWO, Ag, and CuS, facilitating carrier migration and suppressing charge recombination. Similar to the interfacial regulation reported in S-scheme heterojunctions, the Ag-mediated interface modulates charge-transfer behavior and promotes the efficient utilization of photogenerated charge carriers for photocatalytic reactions.^73^ Therefore, the BWO/Ag/CuS heterostructure follows an Ag-mediated all-solid-state Z-scheme charge-transfer mechanism, as proposed in Scheme 2b. The photocatalytic reactions involved in TC degradation and Cr (VI) reduction over the BWO/Ag/CuS photocatalyst are summarized in eqs 7–13.

7 $$\mathrm{BWO}/\mathrm{Ag}/\mathrm{CuS}+\mathrm{h\nu}\to \left(h^{+}\right)\;\mathrm{BWO}/\mathrm{Ag}/\mathrm{CuS}\;(e^{-})$$

8 O2(ad/aq)+e−cb(Cus)→O2−•+2H++2e−cb(CuS)→OH•+−OH

9 H2O+h+Vb(BWO)→H++OH•

10 OH−+h+Vb(BWO)→OH•

11 $$\mathrm{TC}+{h^{+}}_{Vb}\;(\mathrm{BWO})\to \mathrm{degrade}\;\mathrm{product}$$

12 TC+OH•→degradeproduct

13 $$\mathrm{Cr}\;(\mathrm{VI})+e^{-}\to \mathrm{Cr}\;(\mathrm{III})$$

### 3.10 Reusability and Photocatalyst Stability

The stability of a photocatalyst is an important factor for practical applications. To evaluate the reusability of the BWO/Ag/CuS heterostructure, four consecutive photocatalytic cycles were carried out under visible-light irradiation (Figure S15a). The photocatalyst retained most of its activity toward both TC degradation and Cr (VI) reduction after four cycles, indicating good operational stability. The XRD patterns and FESEM images of the recovered catalyst showed no significant changes compared to those of the fresh sample (Figure S15b), suggesting that the crystal structure and hierarchical morphology were largely preserved during the recycling process. Furthermore, ICP–MS analysis revealed only negligible leaching of Bi, Ag, and Cu species after the photocatalytic reaction (Table S1), confirming the good chemical stability of the BWO/Ag/CuS photocatalyst. The slight decrease in photocatalytic activity after repeated cycles is likely due to surface fouling and partial blockage of active sites rather than structural degradation. A comparison of the photocatalytic performance of the present BWO/Ag/CuS catalyst with previously reported photocatalysts for TC degradation and Cr (VI) reduction is provided in Table 1. The BWO/Ag/CuS heterostructure exhibited competitive photocatalytic activity together with good cycling stability, demonstrating its potential for wastewater treatment applications.

**Table 1:** A Comparison of Photocatalytic Degradation of TC or Reduction of Cr (VI) Using Various Reported Bi-Based Photocatalysts and under Visible Light Irradiation

(a) Photodegradation of TC at neutral pH						
Photocatalysts	Concentration (mg/L)	Dosage (g/L)	Time (min)	k (min^–1^)	Active ROS	Refs
Bi_2_WO_6_/MoS_2_	10	0.3	0.0309	90	^•^OH	^19^
BiVO_4_/Ag/Ag_2_CO_3_	20	0.4	0.0180	150	^•^OH	^15^
Bi_2_Sn_2_O_7_/AgI	20	1	50	0.0361	^•^O_2_^–^ & ^•^OH	^14^
Bi_2_WO_6_/g-C_3_N_4_/Ag	15	0.5	60	0.0282	^•^O_2_^–^ & ^•^OH	^18^
Bi_2_WO_6_/Ag/CuS	50	0.5	60	0.0336	^•^O_2_^–^& ^•^OH	This work
″	20	″	30	0.0635	″	″

(b) Photoreduction of Cr (VI) at various pH							
Photocatalysts	Concentration (mg/L)	pH	Dosage (g/L)	Time (min)	k (min^–1^)	Active ROS	Refs
TiO_2_/BiOCl	15	5	1	180	0.0133	^•^OH	^16^
Bi_2_WO_6_/g-C_3_N_4_	10	2	1	120	0.0414	^•^OH	^74^
BiOI/Bi_5_O_7_I	20	2	0.5	60	0.0432	^•^O_2_^–^ & ^•^OH	^17^
BiVO_4_/FeVO_4_@rGO	20	2	0.6	90	0.0274	^•^O_2_^–^ & ^•^OH	^65^
Bi_2_WO_6_/Ag/CuS	40	3.5	0.5	60	0.0322	^•^O_2_^–^ & ^•^OH	This work
″	20	″	″	30	0.0712	″	″

## 4 Conclusions

In this study, a Z-scheme Bi_2_WO_6_/Ag/CuS photocatalyst was successfully fabricated through a combination of photoreduction and solvothermal methods. The incorporation of Ag nanoparticles and CuS onto the Bi_2_WO_6_ surface significantly improved visible-light absorption, photocurrent response, and charge separation efficiency compared with pristine Bi_2_WO_6_ and the binary Bi_2_WO_6_/CuS system. The ternary composite exhibited a photocurrent density approximately 3.87 times higher than that of pure Bi_2_WO_6_. Under visible-light irradiation, the optimized catalyst achieved 92% degradation of TC and 90% reduction of Cr (VI) within 60 min, along with excellent stability and recyclability. The corresponding apparent rate constants for TC degradation and Cr (VI) reduction were 6.72 and 8.25 times higher than those of pristine Bi_2_WO_6_, respectively. Band-structure analysis together with EPR results confirmed an all-solid-state Z-scheme mechanism with the simultaneous generation of ^•^OH and ^•^O_2_^–^ radicals. In the mixed TC/Cr (VI) system, the simultaneous oxidation of TC and reduction of Cr (VI) facilitated efficient utilization of photogenerated charge carriers, thereby enhancing the overall photocatalytic performance. LC–MS analysis further identified the degradation intermediates and proposed the degradation pathway of TC. Overall, the results demonstrate that integrating Ag nanoparticles with Bi_2_WO_6_ and CuS is an effective approach for constructing efficient visible-light-driven photocatalysts for environmental remediation.

## Supplementary Material



## Notes

FESEM images, high-resolution XPS spectra, N_2_ adsorption-desorption, PL spectra, loading of Ag and CuS, and comparison, pH-dependent surface charge profiles, kinetic plots, chromatogram of TC degradation, MS spectra, removal of TC, Cr (VI), their mixtures, Mott-Schottky plots and recyclable studies (PDF)
