# Supplementary material for: Dual-Functional Plasmonic Z‑Scheme Bi2WO6/Ag/CuS Ternary Nanocomposite for Photocatalytic Oxidation of Tetracycline and Reduction of Cr (VI) under Visible Light
Source: ACS Omega. 2026 Sep 18;11(38):57449–63. doi: 10.1021/acsomega.6c05796 (PMC13629063; doi:10.1021/acsomega.6c05796)
Supplement: Supplementary file 1 [file ao6c05796_si_001.pdf]

Supporting Information

Dual-Functional Plasmonic Z-Scheme Bi_2WO_6/Ag/CuS Ternary Nanocomposite for Photocatalytic Oxidation of Tetracycline and Reduction of Cr (VI) under Visible Light

Girija Shankar Jena and Rajakumar Ananthakrishnan*

Department of Chemistry, Green Environmental Materials & Analytical Chemistry Laboratory, Indian Institute of Technology, Kharagpur 721302, India. E-mail: raja@chem.iitkgp.ac.in

Supporting Information

Fig. S1 (a &b) FESEM image of BWO/Ag, and (c) Ag nanoparticles

Fig. S2 FESEM-EDX spectra for BWO/Ag/CuS

Fig. S3 Elemental Mapping Analysis Reveals Uniform Distribution of Bi, O, W, Ag, Cu, and S in the BWO/Ag/CuS nanocomposite.

Fig. S4 High- resolution XPS spectra of S 2s *of* BWO/Ag/CuS composites.

Fig. S5 High- resolution XPS spectra of (a) O 1s, (b) Bi 4f, (c) W 4f of BWO and BWO/Ag/CuS.

Fig. S6 N_2 Adsorption-Desorption Characteristics of BWO and BWO/Ag/CuS Composites.

Fig. S7 PL spectra of BWO, BWO/CuS and BWO/Ag/CuS samples.

Fig. S8 (a) Effect of Ag loading on the photocatalytic degradation of TC over BWO/Ag composites; (b) Effect of CuS loading on the photocatalytic degradation of TC over BWO/Ag/CuS ternary nanocomposites; (c) Comparison of the photocatalytic degradation of TC over the BWO/Ag/CuS heterostructure and its physical mixture; (d) UV–Vis calibration curve of TC.

Fig. S9 (a) pH-dependent surface charge profile of BWO/Ag/CuS, (b) Effect of initial TC concentration; (c) Pseudo-first-order kinetic plots of TC degradation; (d) Effect of coexisting inorganic ions and humic acid (HA); (e) Effect of different water matrices on TC degradation.

Fig. S10 Chromatogram of TC degradation solution at 0 min, 30 min, and 60 min.

Fig. S11 MS spectra of emerging Fragmented intermediates with m/Z from TC solution at 0 min, 30 min and 60 min.

Fig. S12 Kinetic Plot of ln (C_t/C_0) *vs* time for Cr (VI) reduction kinetics using prepared samples BWO, BWO/CuS and BWO/Ag/CuS under visible light irradiation

Fig. S13 Removal of TC and Cr(VI) as single and mixed contaminants over the BWO/Ag/CuS photocatalyst under visible-light irradiation at pH 3.5.

Fig. S14 Mott-Schottky plot of (a) BWO, and (b) CuS

Fig S15. (a) Photocatalytic recyclability of simultaneous TC and Cr (VI) removal by ternary system. (b) PXRD patterns and SEM images of BWO/Ag/CuS before and after four times cycles.

Chemicals required:

The chemicals Bismuth nitrate (Bi $(NO_3)_3{\cdot}5H_2O$), Sodium tungstate dihydrate ($Na_2WO_4{\cdot}2H_2O$), Ethylene glycol ($C_2H_2O_6$), Copper nitrate trihydrate (Cu $(NO_3)_2{\cdot}3H_2O$), sodium thiosulfate pentahydrate ($Na_2S_2O_3$), and $AgNO_3$ and DMPO were obtained from Tokyo Chemical Industry (India). All chemicals and solvents used in this study were of analytical grade with a purity of 99.9%.

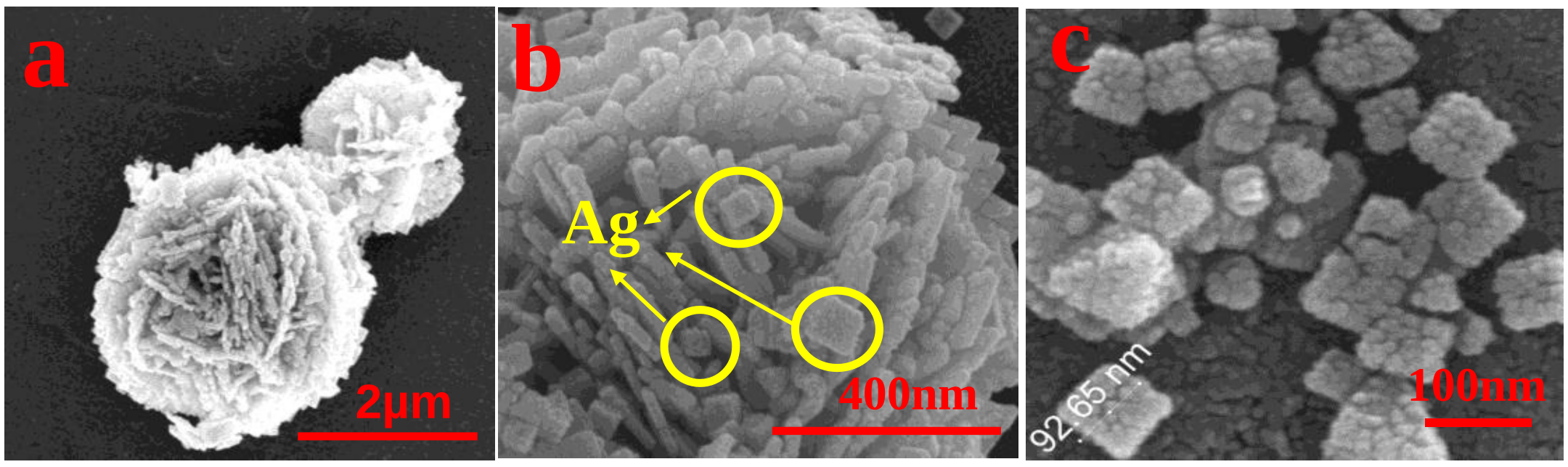

Fig. S1. (a &b) FESEM image of BWO/Ag, and (c) Ag nanoparticles

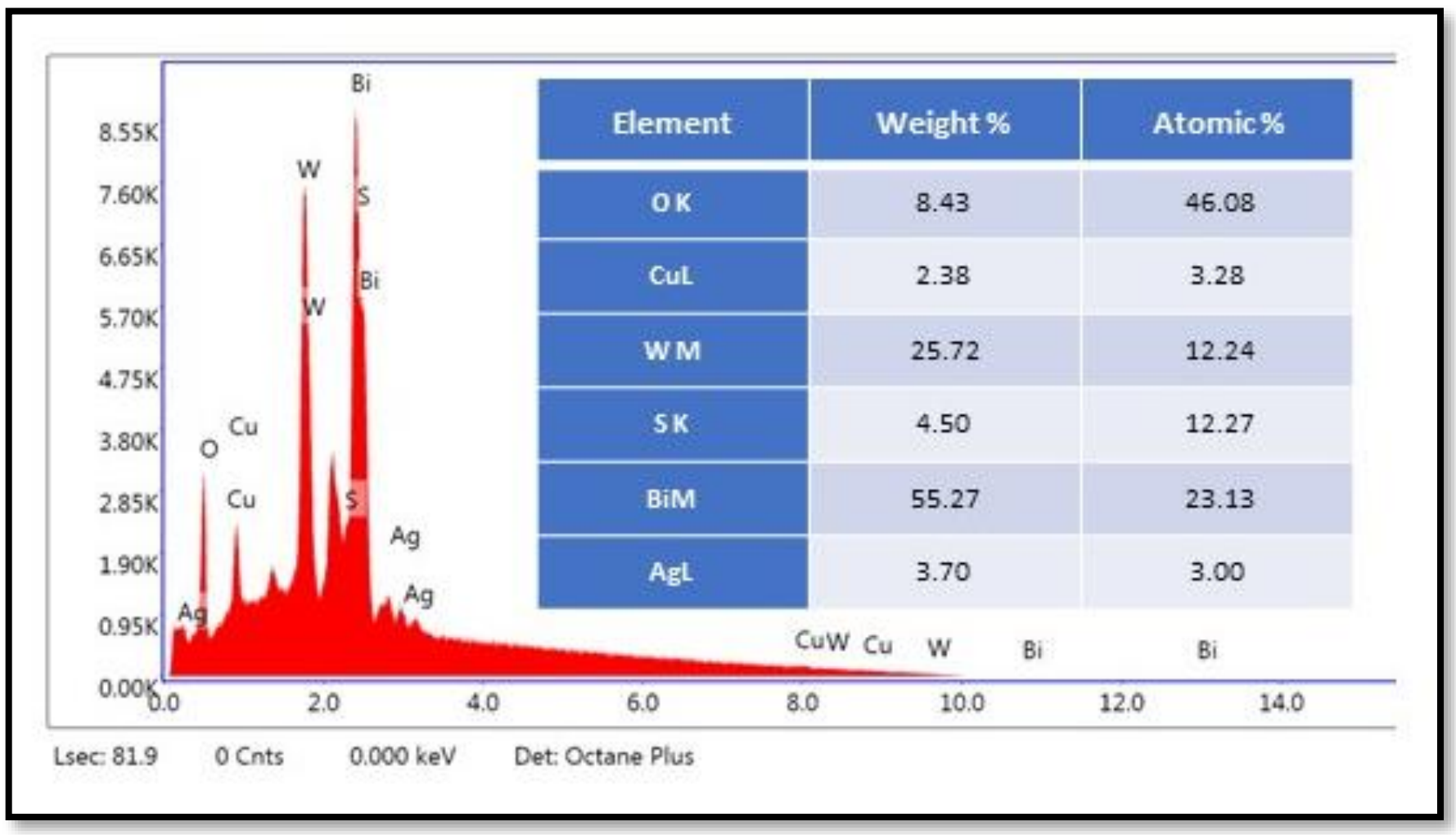

Element	Weight %	Atomic %
O K	8.43	46.08
CuL	2.38	3.28
W M	25.72	12.24
S K	4.50	12.27
BiM	55.27	23.13
AgL	3.70	3.00

Fig. S2. FESEM-EDX spectra for BWO/Ag/CuS

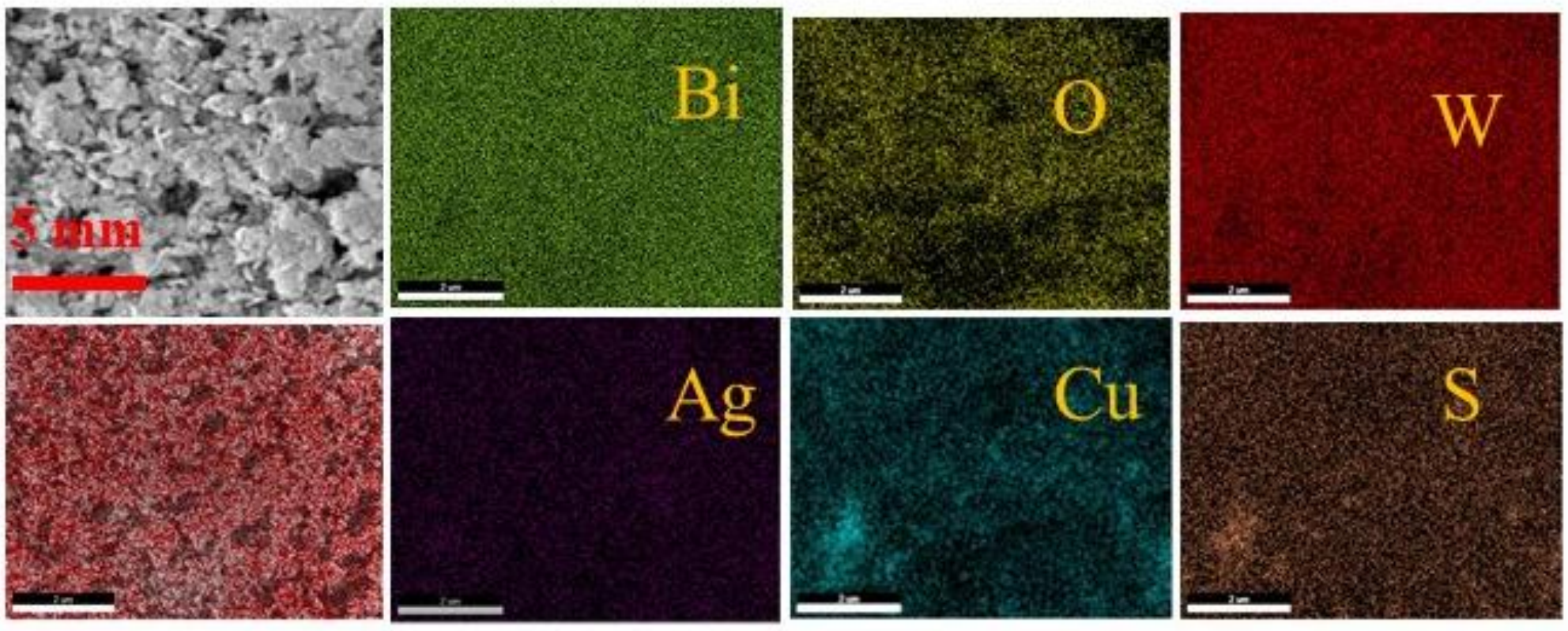

Fig. S3 Elemental Mapping Analysis Reveals Uniform Distribution of Bi, O, W, Ag, Cu, and S in the BWO/Ag/CuS nanocomposite.

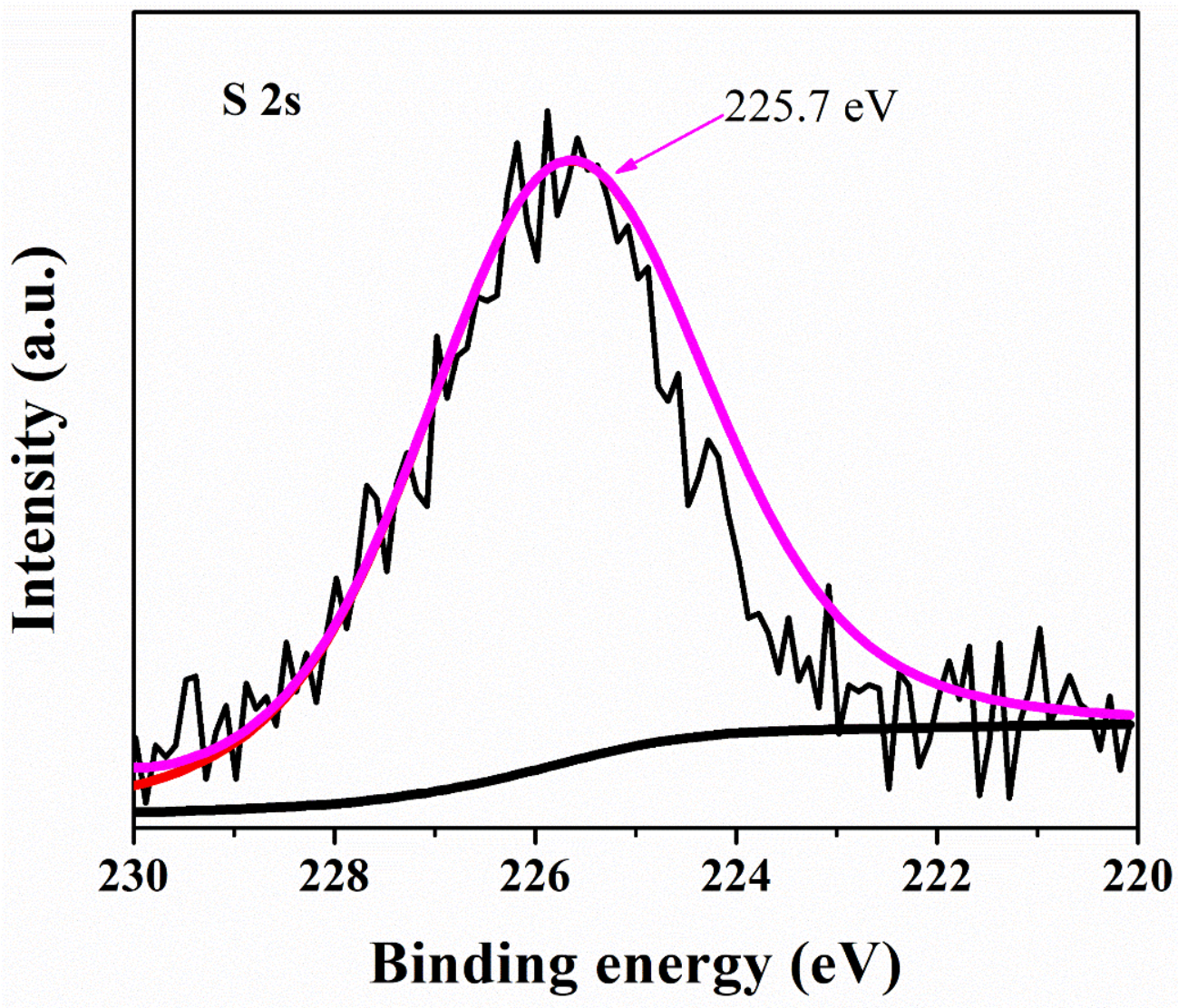

Fig. S4 High- resolution XPS spectra of S 2s of BWO/Ag/CuS composites

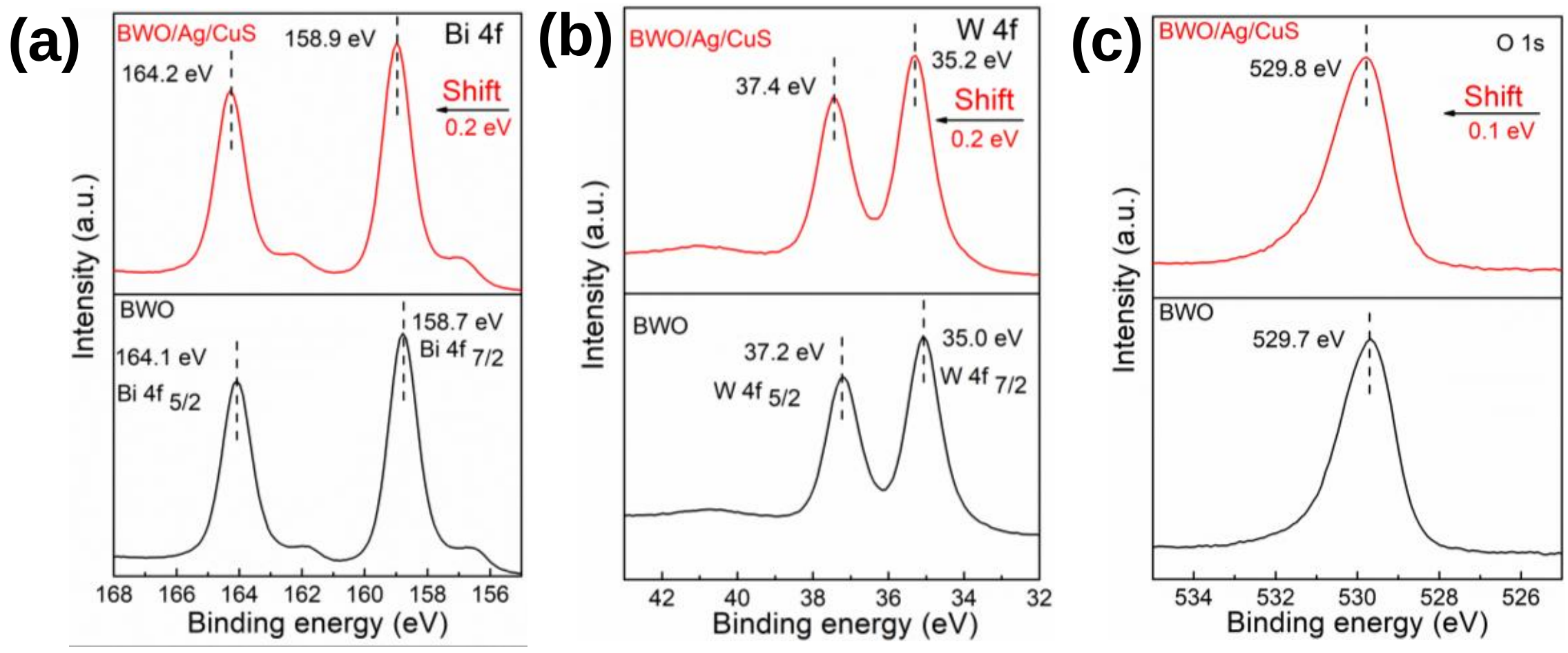

Fig. S5 High- resolution XPS spectra of (a) O 1s, (b) Bi 4f, (c) W 4f of BWO and BWO/Ag/CuS.

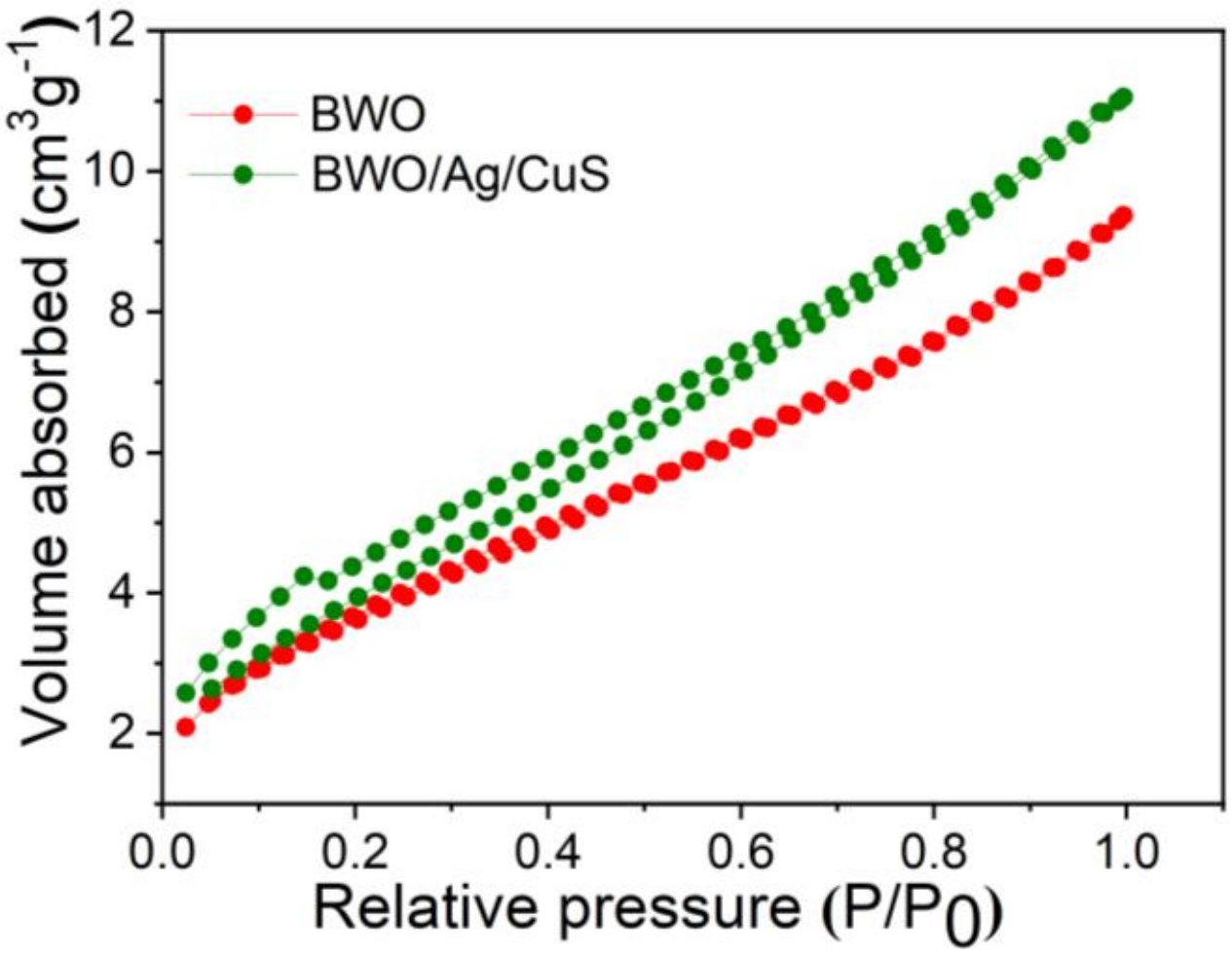

Fig. S6 N_2 Adsorption-Desorption Characteristics of BWO and BWO/Ag/CuS Composites.

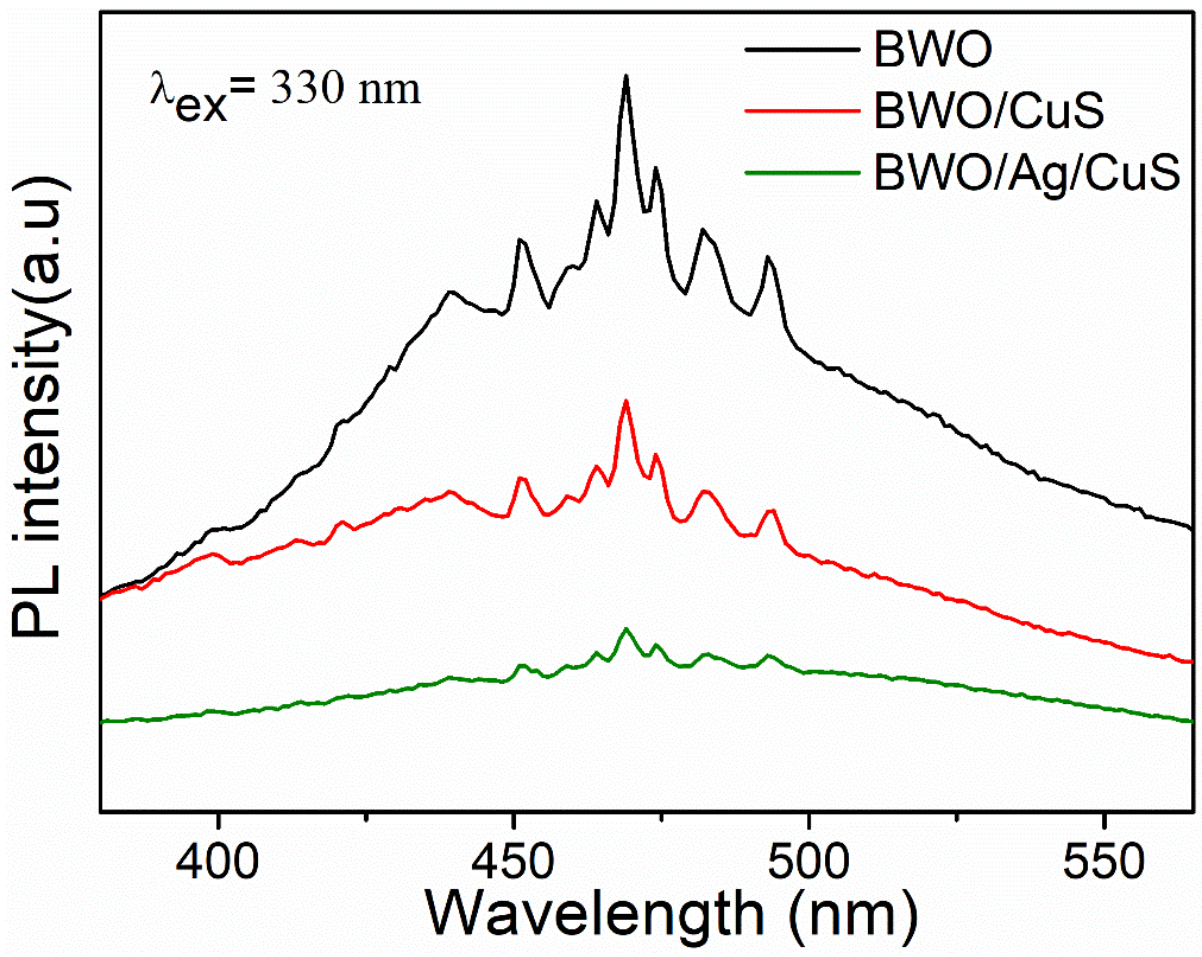

Fig. S7 PL spectra of BWO, BWO/CuS and BWO/Ag/CuS samples

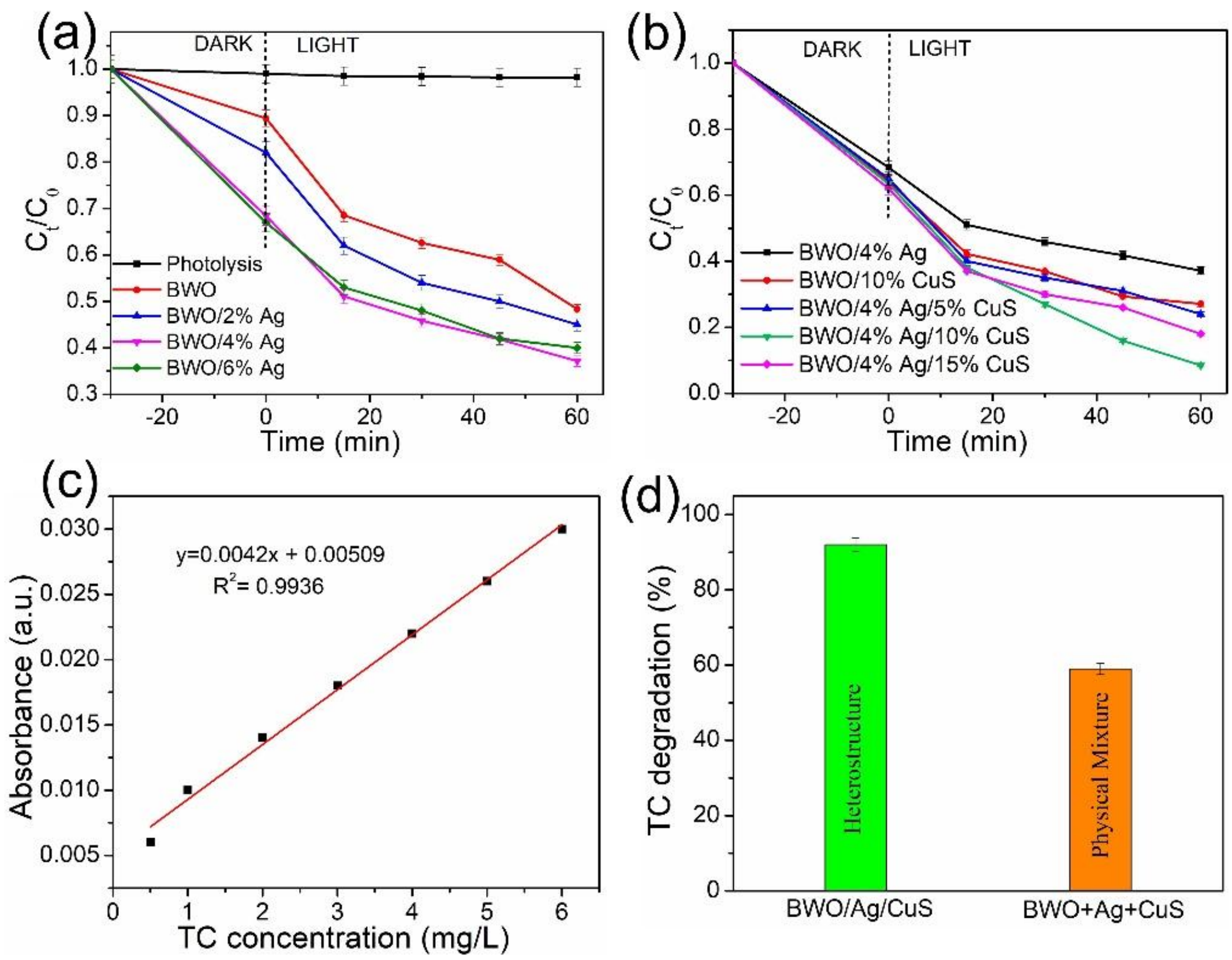

Fig. S8 (a) Effect of Ag loading on the photocatalytic degradation of TC over BWO/Ag composites, (b) Effect of CuS loading on the photocatalytic degradation of TC over BWO/Ag/CuS ternary nanocomposite, (c) UV–Vis calibration curve of TC and (d) Comparison of the photocatalytic degradation of TC over the BWO/Ag/CuS heterostructure and its physical mixture.

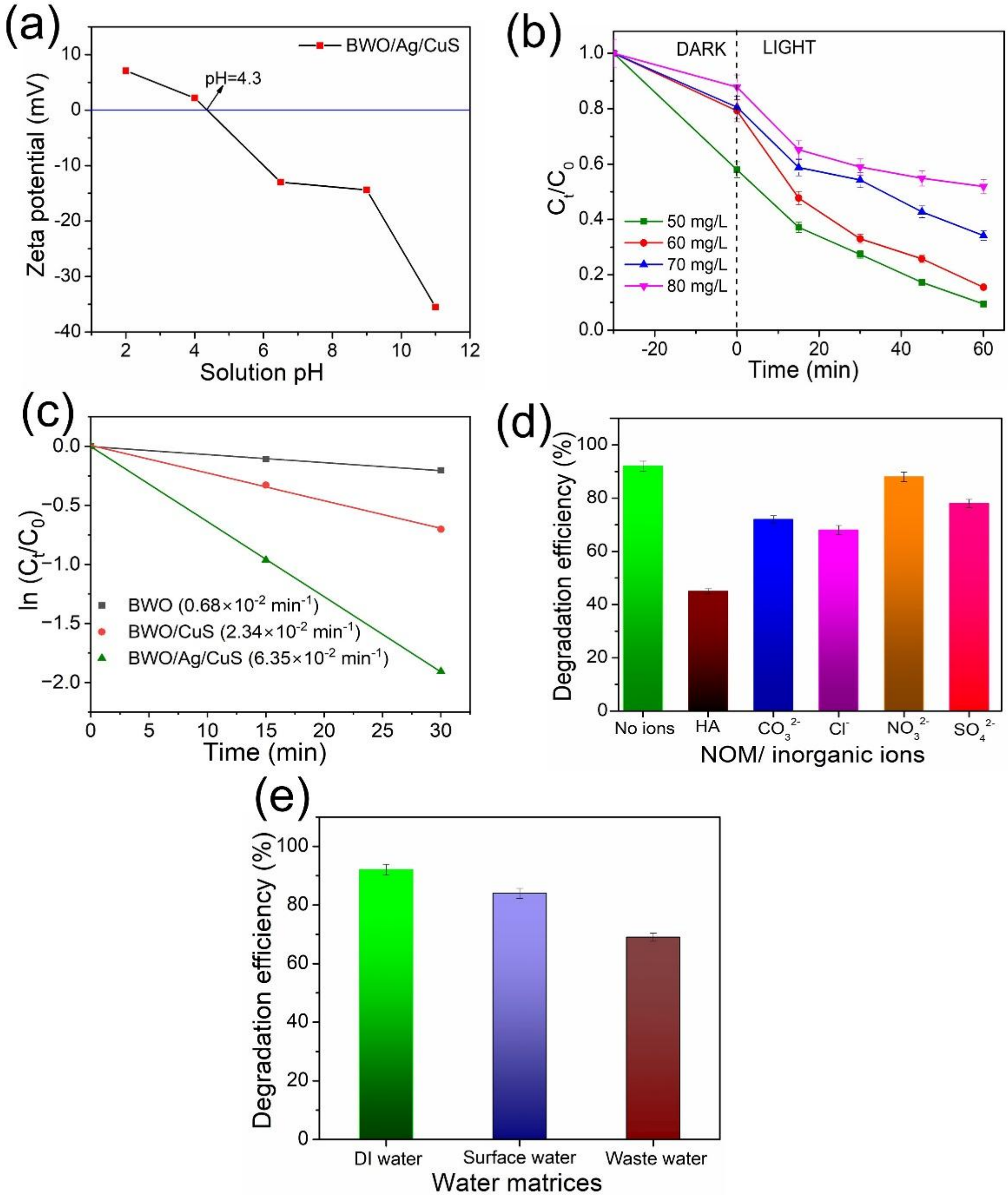

Fig. S9 (a) pH-dependent surface charge profile of BWO/Ag/CuS, (b) Effect of initial TC concentration, (c) Pseudo-first-order kinetic plots of TC degradation, (d) Effect of coexisting inorganic ions and humic acid (HA), and (e) Effect of different water matrices on TC degradation.

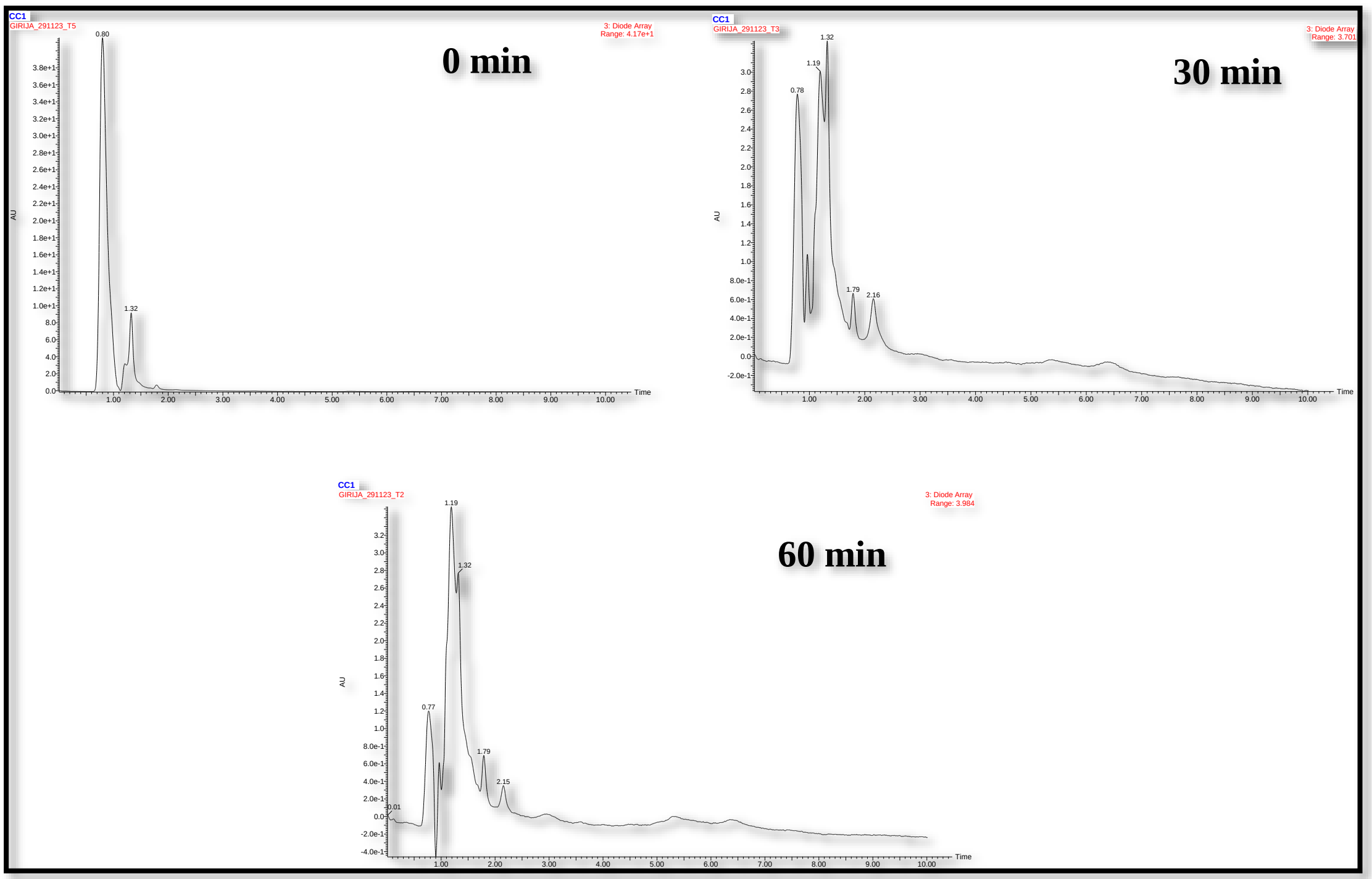

Fig. S10 Chromatogram of TC degradation solution at 0 min, 30 min, and 60 min.

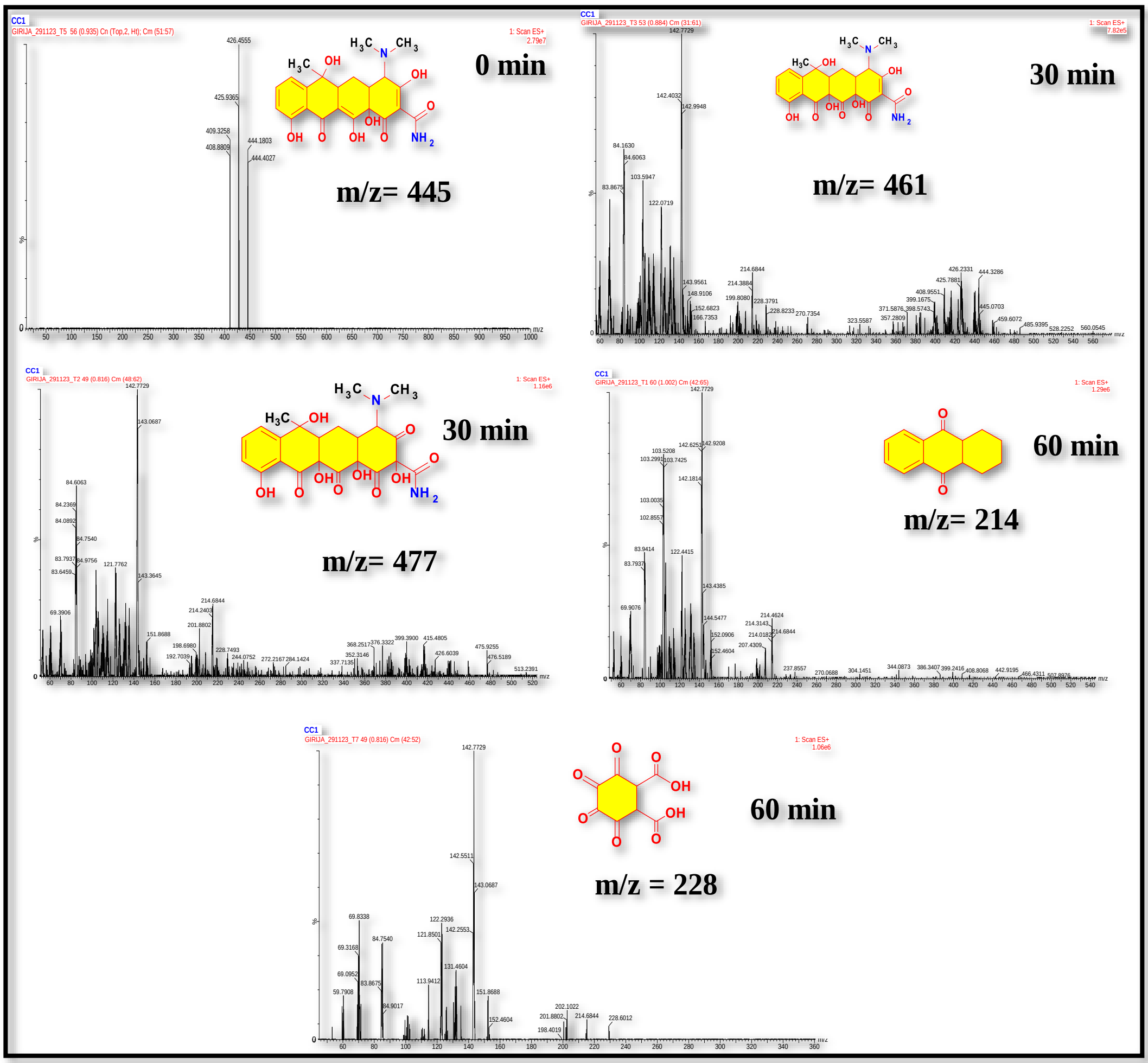

Fig. S11 MS spectra of emerging Fragmented intermediates with m/Z from TC solution at 0 min, 30 min and 60 min.

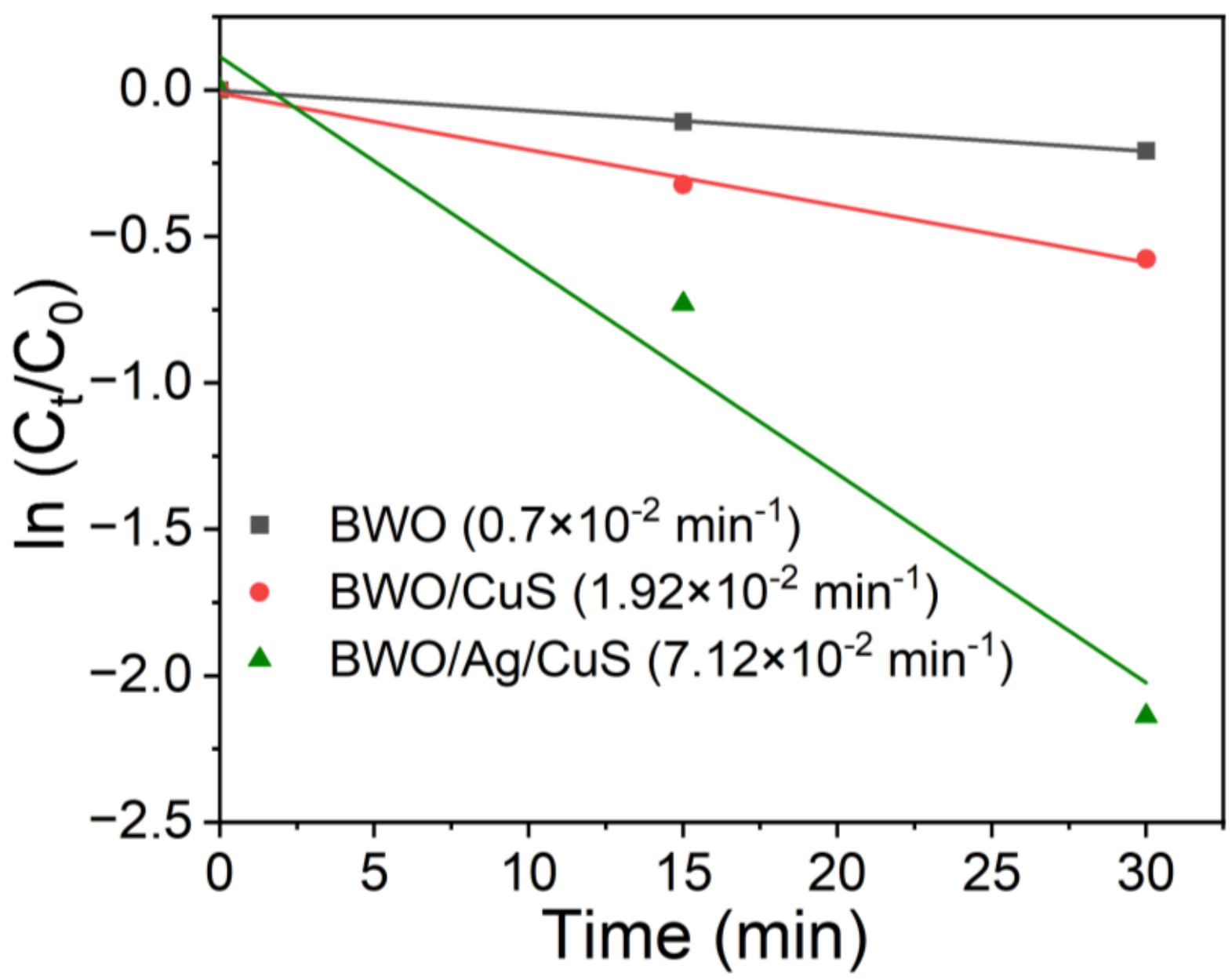

Fig. S12 Plot of ln (C_t/C_0) *vs* time for Cr (VI) reduction kinetics using prepared samples BWO, BWO/CuS and BWO/Ag/CuS under visible light irradiation.

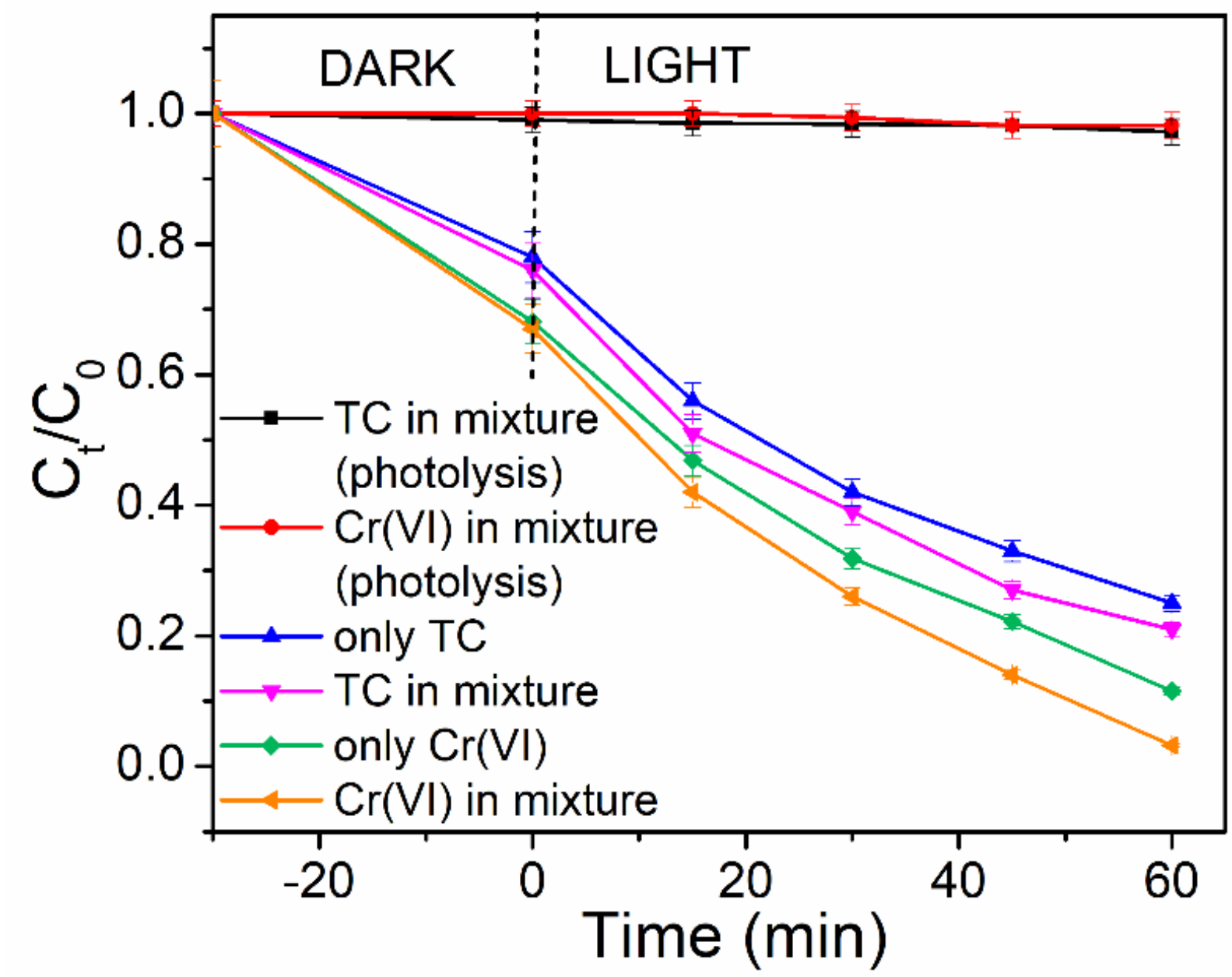

Fig. S13 Removal of TC and Cr (VI) as single and mixed contaminants over the BWO/Ag/CuS photocatalyst under visible-light irradiation at pH 3.5.

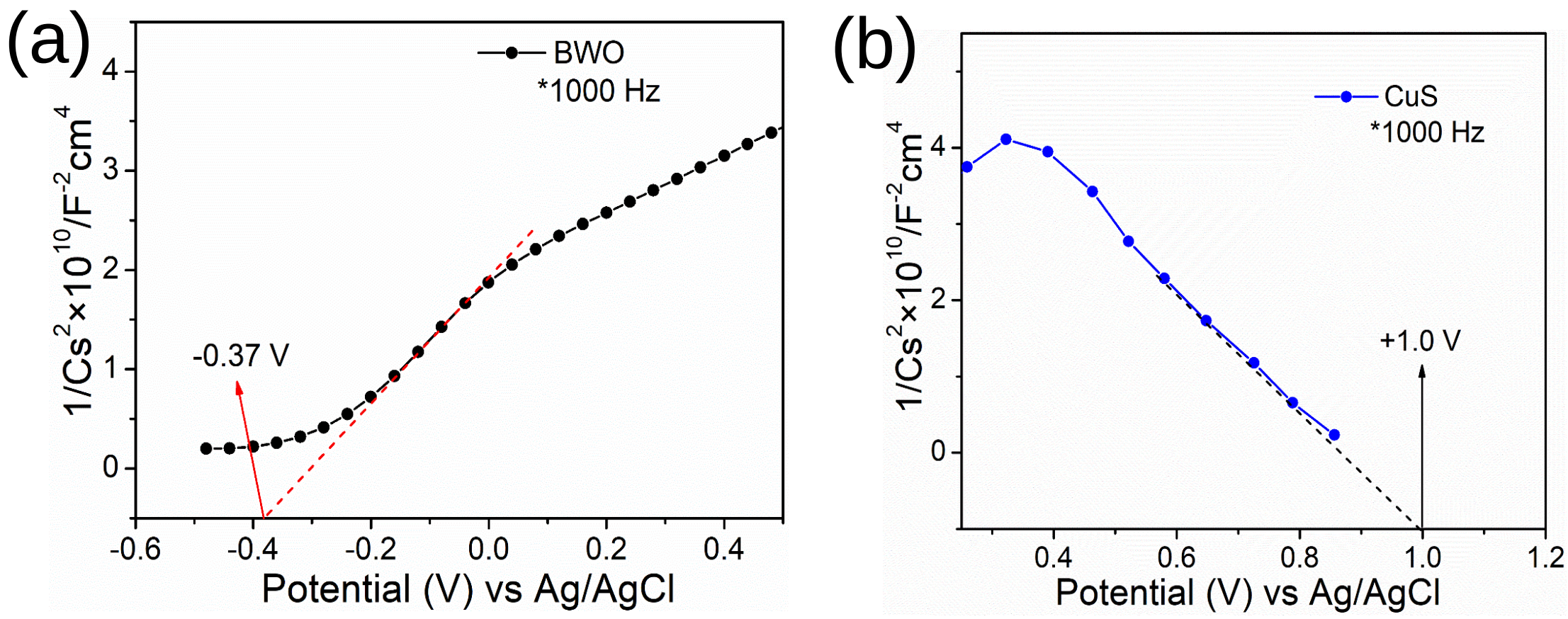

Fig. S14 Mott-Schottky plot of (a) BWO, and (b) CuS

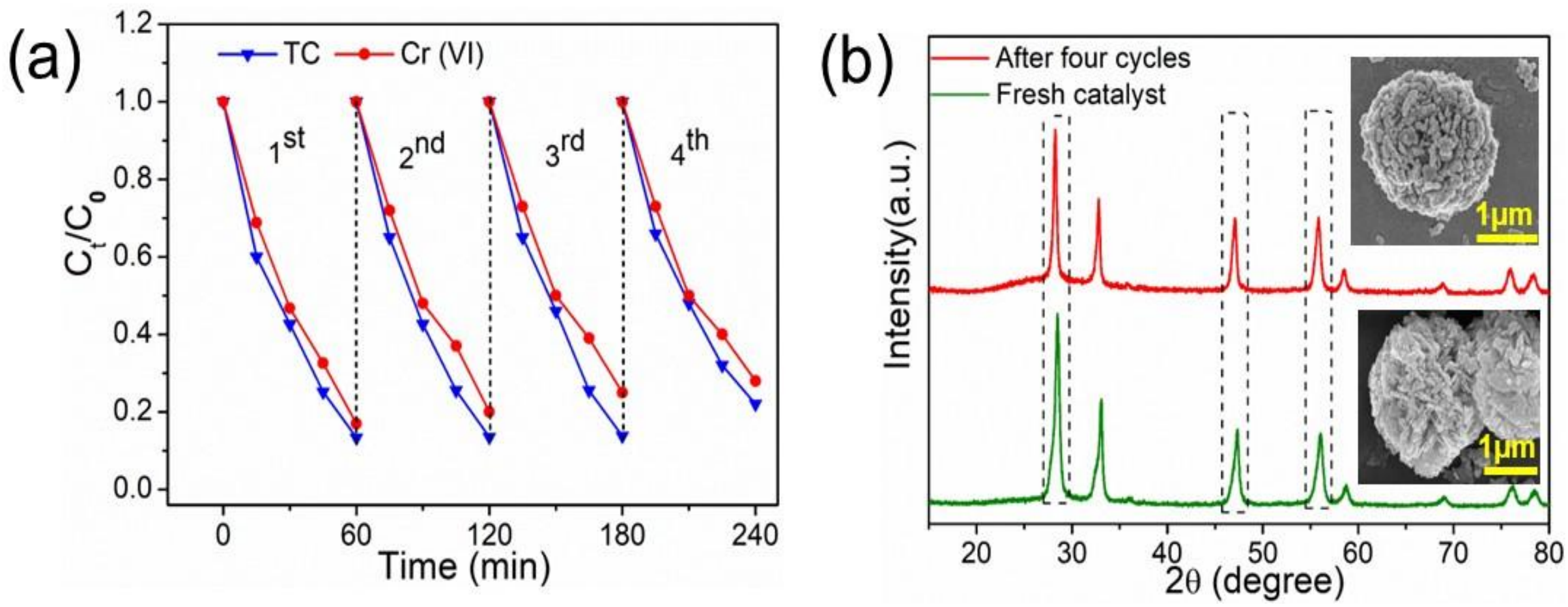

Fig S15. (a) Photocatalytic recyclability of simultaneous TC and Cr (VI) removal by ternary system. (b) PXRD patterns and SEM images of BWO/Ag/CuS before and after four times cycles.

Table S1. ICP–MS analysis of Bi, Cu, and Ag leaching after the photocatalytic reaction.

S. No.	Elements	Concentration (ppb)
1	Bi	40.7807
2	Cu	8.2367
3	Ag	2.8348
